# Estimation of forest above-ground biomass based on stacked ensemble model in Chongqing, China

**DOI:** 10.3389/fpls.2025.1657170

**Published:** 2025-11-07

**Authors:** Jinlian Liu, Zhiyun Chen, Bangxiang Luo, Ao Sun, Xuezhong Wen, Tongyi Huang

**Affiliations:** Institute of Forestry Big Data Application Research, Chongqing Academy of Forestry Planning and Design, Chongqing, China

**Keywords:** above-ground biomass, national forest inventory, remote sensing, machine learning, stacked ensemble model

## Abstract

Accurate regional-scale estimation of forest aboveground biomass (AGB) is critical for effective forest management and terrestrial carbon cycle research. However, applications integrating multiple machine learning models (MLs) for forest AGB estimation in mountainous forests remain limited. In this study, we introduced a practical method to estimate diameter at breast height (DBH < 5 cm) for under-threshold trees using National Forest Inventory (NFI) data. By combining Sentinel-2 remote sensing imagery and DEM data, we employed individual MLs (RF, XgBost, CatBoost and SVM) and a stacking approach to estimate forest AGB in Chongqing under two scenarios: with and without under-threshold trees. The DBH estimation method achieved high accuracy (R²=0.93, RMSE=1.46 cm). Feature importance analysis showed spectral bands dominated predictors, while vegetation and topographic indices varied across models. CatBoost outperformed RF and XgBoost in both scenarios. The stacked ensemble model demonstrated best performances in including under-threshold trees in cross-validation (CV) and external verification (EV) (R²=0.65, RMSE=24.34 Mg·ha ^-^¹; R²=0.68, RMSE=25.45 Mg·ha ^-^¹), generating 10m-resolution AGB maps with consistent spatial patterns suitable for mountainous urban terrain. This work advances AGB estimation in southwestern China’s mountains regions and provides insights for forest ecology and management.

## 1 Introduction

Forests play a crucial role in ecosystem services by providing renewable materials and energy, maintaining biodiversity, conserving water and preventing soil erosion. They also significantly contribute to the global carbon cycle, with plant photosynthesis accounting for approximate 80% carbon storage of the terrestrial ecosystem (Liu et al., 2021). As an important determinant of plant light use, turn over, and respiration, forest above-ground biomass (AGB) is a key index to assess forest maturity and carbon sequestration capacity (Hao et al., 2020; Shen et al., 2020).

Forest AGB estimation approaches can be categorized into filed measurements, remote sensing-based modeling and process-based model simulation (Thornton et al., 2002; Zhu et al., 2020; Guo et al., 2023). Forest inventory data provides a direct measurement which is a valuable resource for forest AGB research, however, it is expensive and time-consuming to implement and limited in spatial explicit mapping. Process-based models contain detailed ecological processes and simulate biomass allocation dynamics which can be used for AGB estimation, but in most cases, running these models is a daunting task since they need abundant variables and field specific calibrations (Veroustraete et al., 2002; Tian et al., 2017). Due to the limitations of traditional forest inventory methods and process-based model simulations, remote sensing-based approaches has been widely employed in forest AGB in the past decades (Goetz et al., 2009; Zhu et al., 2020), with remote sensing technology achieving considerable progresses and numerous agencies launching multi-sensor satellites (Qian et al., 2021). Accurate forest AGB estimation via remote sensing depends on three factors: field data, imagery acquisition, and model selection (Puliti et al., 2020; Wai et al., 2022; Feng et al., 2025).

Field data used for remote sensing-based AGB estimation typically comes from three sources: plots measured by researchers for specific studies, national forest inventory (NFI) data, and data compiled from previously published literature. Data from literature is often used as indirect validation data rather than a direct data source of research (Chang et al., 2021). Field data collection offers flexibility in plot design (density, size) and measurement thresholds to meet specific research objectives; however, this approach may be impractical for studies conducted over large geographical extents (Lei et al., 2009; Li et al., 2018; Chen et al., 2019a). NFI data was conducted through periodic surveys of permanent sample plots to monitor the status of forest resources at national scale. NFI data format is consistent at the provincial scale, making it a valuable source for regional remote sensing-based AGB assessments (Lei et al., 2009; Xie et al., 2011). However, using NFI data without any pre-processes in remote sensing-based AGB studies could introduce uncertainties due to its measurement threshold and sub-sample design in NFI field measurements (Fridman et al., 2014; Breidenbach et al., 2020; Perssion and Ståhl, 2020; Wang et al., 2024). For instance, China’s National Forest Inventory adopts a 5-cm DBH measurement threshold, excluding smaller trees per technical specifications. This protocol stems from cost-benefit analyses indicating that sampling sub-threshold trees incurs disproportionate resource expenditures relative to their marginal utility in national-scale forest ecology assessments and management frameworks. Nevertheless, this exclusion potentially underestimated the ecological significance of under-threshold trees (DBH < 5 cm) in forest ecosystems, considering natural regeneration dynamics in certain regions and the impact of China’s ongoing afforestation and forest quality improvement initiatives (Xie et al., 2011; Li et al., 2022).

Remote sensing data used for forest AGB estimation is typically categorized into radar and optical data. Radar data, with variations in wavelength, polarization, and angle of incidence, had been proven useful for forest biomass estimation due to their influence on backscatter coefficients (Goetz et al., 2009). However, small bandwidth, high cost of airborne acquisition, low sampling density and limited coverage hinder its application in regional forest AGB research (Lu, 2006; Kumar et al., 2015). Optical sensors, including Sentinel-2, Landsat-8, SPOT, ASTER, CVERS, QuickBird, MODIS and AVHRR, have also been primary data sources for forest AGB estimation with various spectral, spatial, radiometric and temporal resolutions (Puliti et al., 2020; Yang et al., 2023). In recent years, Sentinel-2 and Landsat-8 have emerged as widely used optical remote sensing platforms for forest AGB estimation at regional scales, owing to their free access and multi-spectral capacity (Puliti et al., 2020; Qian et al., 2021; Zhu et al., 2020). Compared with Landsat-8, Sentinel-2 offers higher spatial and temporal resolution, as well as broader spectral band range. Among optical remote sensing data, Sentinel-2 includes three bands in the red-edged range, which are particularly useful for monitoring vegetation health information, making it popular in forest AGB research. The procedure for estimating forest AGB using Sentinel-2 typically involved extracting reflectance, vegetation and biophysical indices from images, and then building the relationship between these variables and forest AGB values (Chrysafis et al, 2017; Babcock et al, 2018; Chen et al., 2019a). Each index adds certain information about forest AGB. Band reflections, such as red, green, infrared, red-edge bands, differentiate ground objects and reflect vegetation growth. Compared to visible bands, the red-edge band is highly responsive to minor changes in vegetation canopy structure and chlorophyll contents (Wai et al., 2022). Vegetation indices, such as the normalized difference vegetation index (NDVI) and Normalized Difference index (NDII), are simple and effective to evaluate surface vegetation status and have been widely employed in forest AGB estimation. However, the spectral saturation effect in areas of high vegetation density could affect model accuracy. Some optimized indices, such as the simple ratio (SR), could overcome the saturation effect due to their greater sensitivity to higher AGB values (Schlerf et al., 2005). Biophysical indices, such as leaf area index (LAI), fraction of green vegetation cover (FCOVER) and Chlorophyll content in the leaf (Cab), provide detailed information on vegetation spatial distribution and dynamics and thus improve forest AGB estimation performance (Zhang et al., 2023). Additionally, topographic features, such as elevation, slope and aspect, are closely related to forest growth and distribution pattern. Variables obtained from the high-resolution DEM data further aids AGB estimation and significantly influence the spatial distribution of estimated AGB map (Chen et al., 2019b; Wang et al., 2021). Thus, combining information of different indices is important to improve forest AGB estimation accuracy.

Forest AGB estimation employs two modeling frameworks: parametric models (regression with predefined function forms) and non-parametric approaches (machine learning algorithms without distribution assumptions). Typically, parametric models are divided into two groups. The first category comprises of linear and non-linear models that calculate the relevance of remote sensing variables to forest AGB, such as stepwise regression models (SWR), logistic regression and correlation coefficient analyses (Lu, 2005; Liu et al., 2017; Ma et al., 2021). The other category consisted of spatial co-simulation algorithms that spatially interpolate forest AGB between remote sensing variables and plot data, such as geographically weighted regression (GWR) and sequential Gaussian simulation (Zhang et al., 2013; Chen et al., 2018). Parametric models heavily depend on measurable vegetation parameters, thus, inaccuracies in these parameters inevitably interfere with AGB estimation results. Non-parametric models, also referred as machine learning methods, include k-nearest neighbor (KNN), random forest (RF), extreme gradient boosting (XgBoost), Categorical Boosting (CatBoost), support vector machine (SVM), maximum entropy (MaxEnt), bagging stochastic gradient boosting (BagSGB), etc. (Li Y. C. et al., 2019). Compared to the parametric models, machine learning approaches have the ability to process complex and non-linear relationships, estimate with high precision, and deal with various data types (Puliti et al., 2020; Li et al., 2021; Tang et al., 2022). For example, RF is easier to achieve higher accuracy due to its strong generalization ability, insensitivity to multicollinearity and low sensitivity to noise (Chen et al., 2019b; Li et al., 2020). XgBoost is capable of processing large-scale data, and the sparse perception algorithm automatically learn its splitting direction in the samples with missing eigenvalues without additional preprocessing (Liu et al., 2017). CatBoost has advantages in handling class features, controlling overfitting, dealing with missing values, and computing efficiency (Zhang et al., 2024). SVM excels at handling high-dimensional data and effectively avoids the “curse of dimensionality,” meaning that an increase in the number of features does not necessarily lead to a decline in performance (Luo et al., 2024). Due to the inherent strengths and limitations across different algorithms, no single method has emerged as universally optimal for estimating forest AGB. Empirical studies demonstrate that stacking can significantly improve predictive accuracy by synergistically integrating outputs from multiple single-algorithm models, thereby mitigating information loss. Such stacking frameworks have been successfully applied in diverse domains, including weather forecasting, and environmental monitoring. For instance, a stacked ensemble combining multiple machine learning algorithms with a deep residual network achieved high cross-validation accuracy in generating surface visibility products (Zhang et al., 2024). Furthermore, based on entropy weighting, a composite model is developed by integrating moving average (ARIMA), artificial neural networks (ANNs), and exponential smoothing (ESM) to predict PM2.5 concentration time series (Ma et al., 2021). Despite notable progresses, research on stacked ensemble models for AGB estimation was still limited, revealing significant methodological opportunities.

As a mountainous metropolis, Chongqing’s extensive forest coverage establishes it as a critical regional carbon sink. However, slope-driven environmental vulnerability and recurrent drought events pose challenges to carbon pool stability, underscoring the urgent need for further forest AGB research. Consequently, the primary objectives of this study are to: (1) develop a method to estimate DBH of under-threshold trees in 2017 and calculate the plot-level forest AGB values; (2) establish individual and stacked ensemble model for forest AGB estimation and compare their performances; and (3) generate a high spatial resolution forest AGB map for the study area and analyze its spatial distribution.

## 2 Materials and methods

### 2.1 Study area

**Figure 1:**
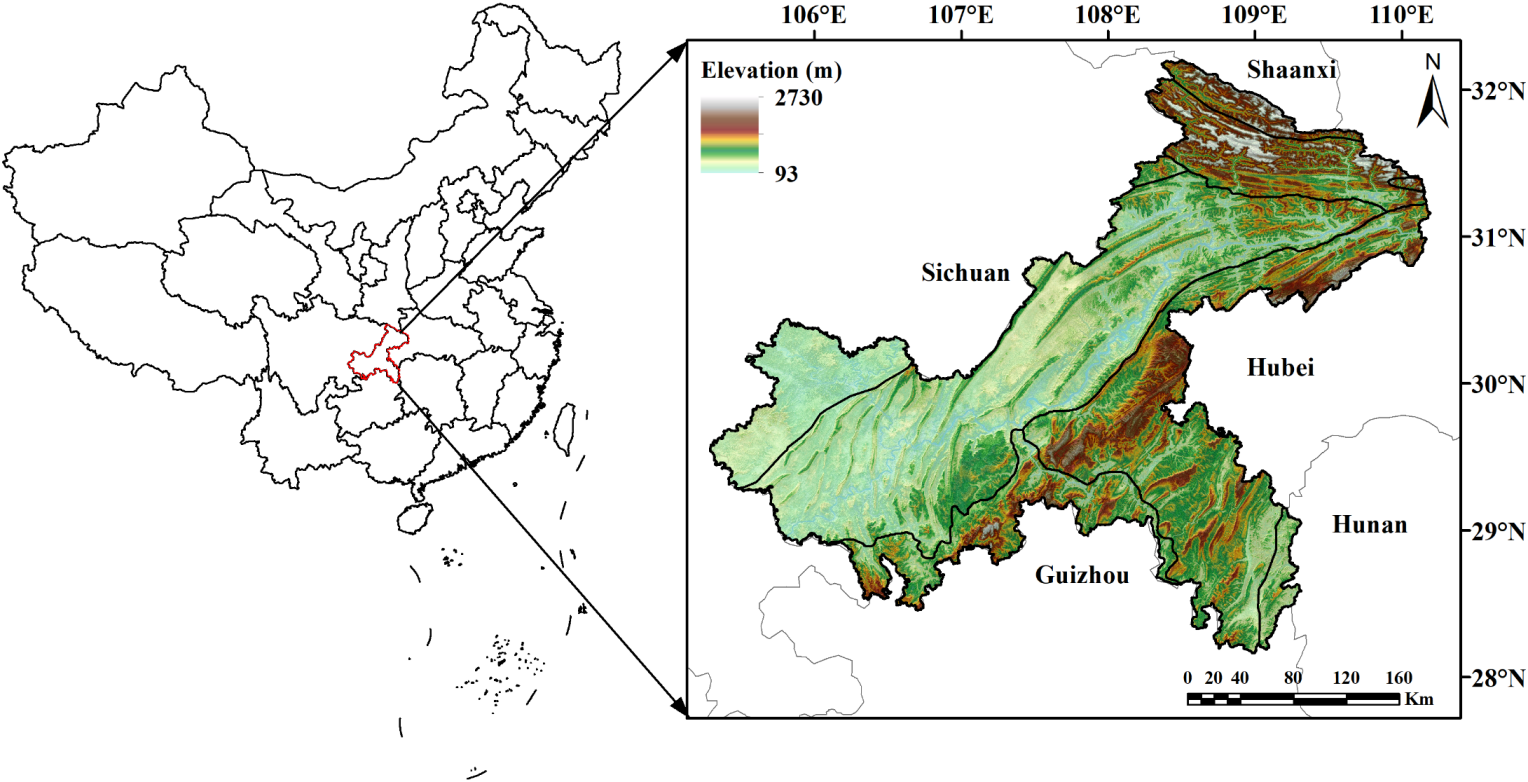
The location and elevation of Chongqing.

Chongqing, located in the upper reaches of the Yangtze River basin, is one of the economy centers in southwest China (**Figure 1**). It spans between 105°28’-110°19’E longitude and 28°16’-32°20’N latitude, covering an area of approximately 82,400 km^2^. Renowned as ‘The Mountain City’, Chongqing is defined by its rugged terrain of hills and mountains. The region falls in the humid subtropical zone, experiencing a typical continental monsoon climate. The mean annual temperature ranges from 17 to 18.8°C, with an average annual precipitation of 1000–1400 mm and annual sunshine duration of 1000–1400 hours. The forest types in Chongqing comprise mainly of evergreen broad-leaved forests, secondary and warm coniferous forests, bamboo forests, and evergreen broad-leaved shrubs. The major tree species found in Chongqing include *Pinus massoniana*, *Cunninghamia lanceolata*, *Cypress*, and *Quercus* spp.

### 2.2 Data source and processing

#### 2.2.1 Field data

The field campaigns was carried out in Chongqing from April to October 2017, according to the NFI technique protocol. Mechanical sampling method was used to set up fixed plots, with a spacing of 4 km × 4 km. Each sample plot was a square of 25.82 m × 25.82 m, covering an area of 0.0667 ha. The tree species, tree number, scale stick type, DBH, volume per tree and age of all trees with DBH > 5 cm and height of dominant trees in the sample plots were measured and recorded. The NFI data also contained the corner coordinates of the sample plot and the horizontal distance between the corner points. After data cleaning, the actual number of available sample plots was 623. In 2021, the NFI data collecting shifted to an annual measurement scheme. This new protocol involved surveying one-fifth of all permanent plots each year, completing a full inventory cycle in five years. Previously, all plots were measured once every five years. Thus, a plot measured in 2017 would have been revisited in either 2021, 2022, or 2023. Consequently, NFI data from 2021 to 2023 could be utilized to estimate the under-threshold DBH from 2017 and delineated plot boundaries based on the coordinates of the four corners.

#### 2.2.2 Sentinel-2 data

Sentinel-2 MSI level 1C products were downloaded from the Copernicus Data Space Ecosystem (https://dataspace.copernicus.eu/) between April and October 2017. Sentinel-2 level 1C data consist of 13 spectral bands at spatial resolutions of 10 m, 20 m and 60 m, respectively. Atmospheric correction was applied using sen2cor (version 2.8.0) to obtain level 2A products. Clouds were masked based on the pixel values of screen classification layer (SCL) products. The 20 m and 60 m images were then mosaic and resampled to 10 m spatial resolution, and the final 12 spectral bands (Band 10 was removed by atmospheric correction) were stored by tiles. 20 cloud-free mosaic tiles were generated based on the UTM zone (48N and 49N) via WGS84 projection which cover the entire study area.

#### 2.2.3 DEM

Digital Elevation Model (DEM) data with spatial resolution of 30 m was downloaded from European Space Agency (https://www.esa.int) and clipped based on Sentinel-2 tile grids and reprojection to WGS 84 UTM zones 48N and 49N.

#### 2.2.4 Remote sensing indices

In our study, 40 remote sensing-based indices were selected for AGB estimation (**Table 1**). Among them, 33 were obtained from Sentinal-2 data (12 band reflectance, 16 vegetation indices and 5 biophysical indices), 7 topographic indices were derived from the DEM data. The final variables used for model training and prediction were 20. The biophysical indices were computed using SNAP (ESA, Windows 64-bit, version 8.0), the others were computed by R.

**Table 1:** Details of features used for AGB estimation.

Data source	Group	Features	Description
Sentinel-2	Reflectance	Band 1	Reflectance at 442.7 nm
		Band 2	Reflectance at 492.7 nm
		Band 3	Reflectance at 559.8 nm
		Band 4	Reflectance at 664.6 nm
		Band 5	Reflectance at 704.1 nm
		Band 6	Reflectance at 740.5 nm
		Band 7	Reflectance at 782.8 nm
		Band 8	Reflectance at 832.8 nm
		Band 8A	Reflectance at 864.7 nm
		Band 9	Reflectance at 945.1 nm
		Band 11	Reflectance at 1613.7 nm
		Band 12	Reflectance at 2202.4 nm
	Vegetation index	BRI^1^	Browning Reflectance Index, (1/B3-1/B5)/B9
		Chlgreen^2^	Chlorophyll Green, B3/B7
		Chlrededge^2^	Chlorophyll Index Red-Edge, (B9/B5) - 1
		CIgreen^3^	Chlorophyll Index Green, (B9/B3) - 1
		CIrededge^2^	Chlorophyll Red-Edge, B5/B7
		CVI^4^	Chlorophyll vegetation index, B9*B5/(B3^2)
		GNDVI^5^	Green Normalized Difference Vegetation Index, (B7-B3)/(B7+B3)
		MCARI^5^	Modified chlorophyll absorption ration index, [(B5-B4)-0.2*(B5-B3)]*(B5-B4)
		MSI^6^	Moisture Stress Index, B11/B8
		MTCI^5^	Meris terrestrial chlorophyll index, (B6-B5)/(B5-B4)
		MVI^7^	Mid-infrared vegetation index, B9/B11
		NDII^8^	Normalized Difference index, (B8 - B11)/(B8 + B11)
		NDSI^9^	Normalized Difference Salinity Index, (B11 - B12)/(B11 + B12)
		NDVI^5^	Normalized Difference Vegetation Index, (B8 - B4)/(B8 + B4)
		SR^10^	Simple Ratio of 833nm/1649nm, B8/B11
		TM5^11^	Simple Ratio of 1650nm/2218nm, B11/B12
	Biophysical index	Cab	Chlorophyll content in the leaf
		CWC	Canopy Water Content
		FAPAR	Fraction of Absorbed Photosynthetically Active Radiation
		Fcover	Fraction of vegetation cover
		LAI	Leaf Area Index
DEM	Topographical index	Aspect^12^	Aspect of the plot
		Curvature^13^	Curvature of the plot
		Elevation	Elevation of the plot
		Roughness^12^	Surface Roughness of the plot, the difference between the maximum and the minimum value of a cell and its 8 surrounding cells
		Slope^12^	Slope of the plot
		TPI^12^	Topographic Position Index, the difference between the value of a cell and the mean value of its 8 surrounding cells
		TWI^5^	Topographic Wetness Index, TWI=ln[Ac/tan(slope)], where Ac is the catchment area directed to the vertical flow

Merzlyak et al., 2003; ^2^Gitelson et al., 2006; ^3^Gitelson et al., 2003; ^4^Hunt et al., 2011; ^5^Chen et al., 2019a; ^6^Heiskanen, 2006; ^7^Schlerf et al., 2005; ^8^Main et al., 2011; ^9^Richardson et al., 2002; ^10^Shibayama et al., 1999; ^11^Datt, 1999; ^12^Wilson et al., 2007; ^13^McNab, 1993.

### 2.3 Method

#### 2.3.1 DBH estimation of under-threshold trees

**Figure 2:**
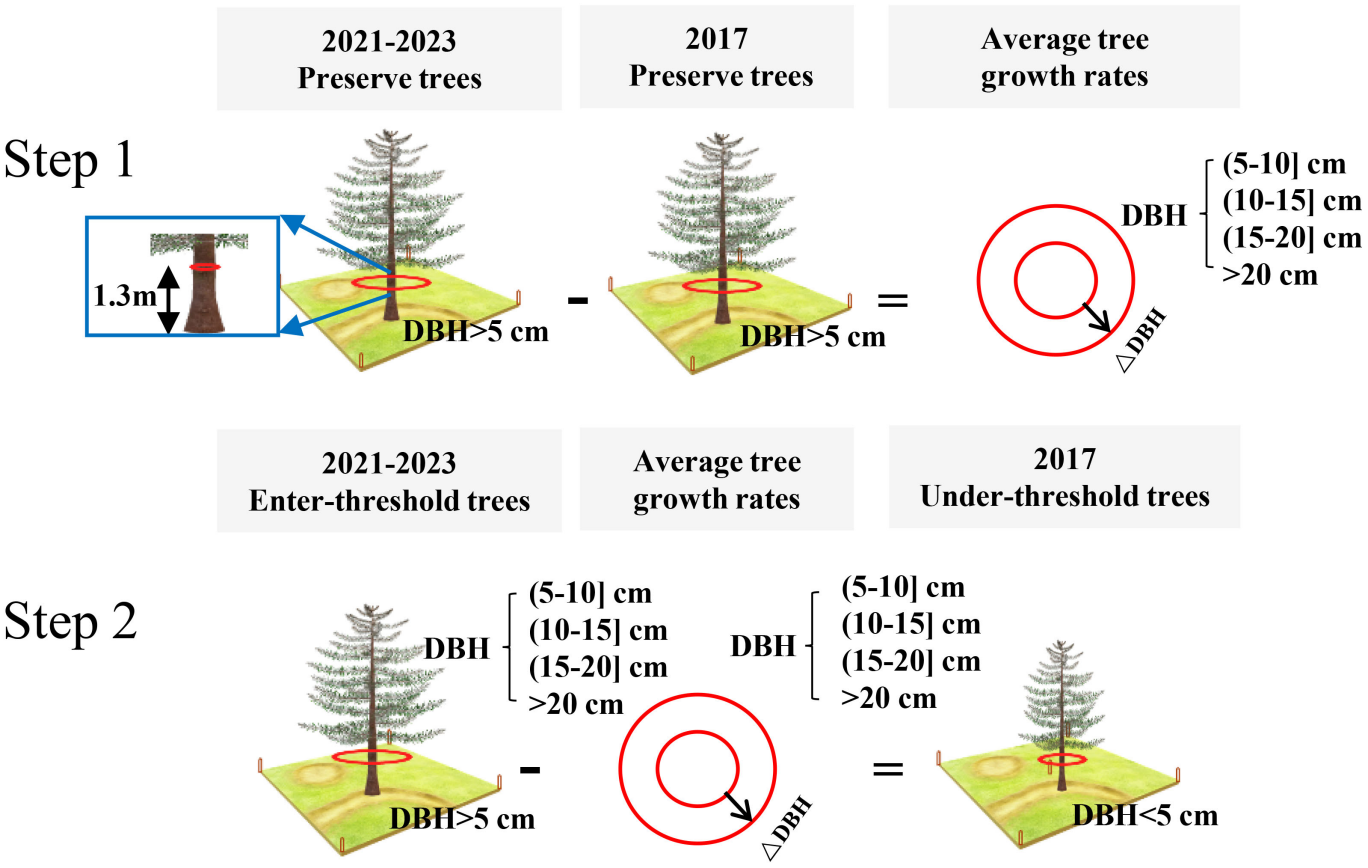
The process of DBH estimation of under-threshold trees. Take a tree species in a plot as an example.

**Figure 3:**
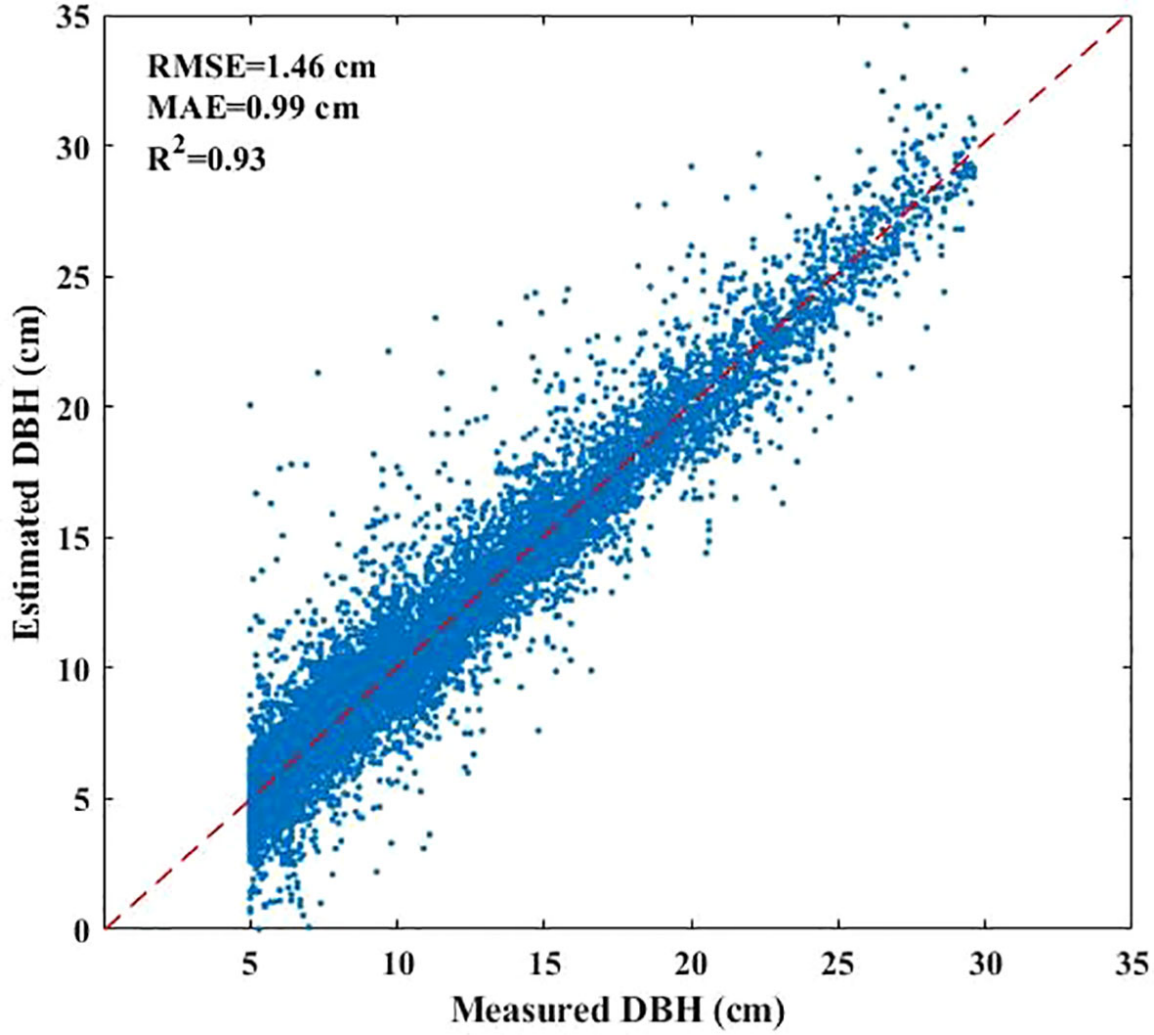
Scatter plot of estimated DBH and measured DBH.

The DBH values of under-threshold trees (DBH < 5 cm) in the 2017 NFI data were estimated based on all preserve trees which DBH reached 5 cm in previous years and enter-threshold trees which DBH reached 5 cm in current forest inventory year data from the 2021–2023 NFI data (**Figure 2**). We first calculated the DBH growth rates of 80% preserve trees in the plots according to the tree ID, and then estimated the average tree growth rates which are grouped by plots, tree species and DBH levels including 4 types as (5-10] cm, (10-15] cm, (15-20] cm and greater than 20cm. The estimated DBH of the remaining 20% preserve trees was compared with the measured DBH to verify the method performance. The result showed that the method has a sound result and can be employed for further analysis (**Figure 3**). Finally, we applied the method to calculate the DBH growth rates for all preserve trees, then these growth rates in different groups were subtracted from entered-threshold trees’ DBH values (same group method) in the 2021–2023 NFI data to get these trees’ DBH values in 2017 when they were under-threshold.

#### 2.3.2 Plot-level AGB calculation

Plot-level AGB was calculated by adopting Zeng and Tang’s method (Zeng and Tang, 2011). Zeng and Tang (2011) compared their approach with different empirical studies across the globe, and demonstrated that the theoretical parameter value of 7/3 is capable of describing the average allometric relationship between AGB and DBH of different tree species in their study. The coefficient between AGB and DBH can be obtained approximately by multiplying 0.3 to wood density ρ. The calculation follows Equation 1.

(1) $$\text{AGB}=0.3\times \text{ρ}\times {\text{D}}^{\frac{7}{3}}$$

where ρ represents basic wood density (g·cm^-3^) and D represents DBH. Plot-level AGB is the sum of the AGB of all the trees in the plot. The ρ used for AGB calculation of different tree species are presented in **Table 2** and **Supplementary Table S1**. The ρ of most tree species refer to Zeng (2018) paper, while ρ of other trees species which are not covered in the paper were adopted from “Testing basic wood density of national dominant species (group)” (China’s Forestry Industry Standard, LY/T 3256-2021).

**Table 2:** Basic wood density (ρ) of different tree species used for plot-level AGB calculation.

Tress species/Groups	ρ (g·cm^-3^)	Tress species/Groups	ρ (g·cm^-3^)
*Quercus*	0.5762	*Tsuga*	0.4420
*Abies*	0.3464	*P.sylvestris* var. *mongolica*	0.3750
*Picea*	0.3730	*Liquidambar formosana*	0.5035
*Betula*	0.4848	*P.elliottii*	0.4118
*Larix*	0.4059	*Salix*	0.4410
*Cunninghamia Lanceolata*	0.3098	*Eucalyptus*	0.5820
*P.massoniana*	0.4476	*Cryptomeria*	0.3493
*Populus*	0.4177	*Robinia pseudoacacia*	0.0674
*P.yunnanensis*	0.3499	*Paulownia*	0.2370
*P.densata*	0.4720	*Keteleeria fortunei*	0.4485
*Tilia*	0.3200	*Phoebe zhennan*	0.4807
*Fraxinus mandshurica*	0.4640	*Cinnamomum*	0.4600
*Juglans mandshrica*		*Sassafras*	
*Phellodendron amurense*		*Phoebe*	
*Schima superb*	0.5563	*Abrus precatorius*	0.5843
*P.tabulaeformis*	0.4243	*Melia azedarach L.*	0.4389
*P.koraiensis*	0.3130	Other pines	0.4500
*Ulmus*	0.4580	Other hardwood broad-leaves	0.6250
*P.armandii*	0.3930	Other softwood broad-leaves	0.4430

#### 2.3.3 Feature selection and variable importance

**Figure 4:**
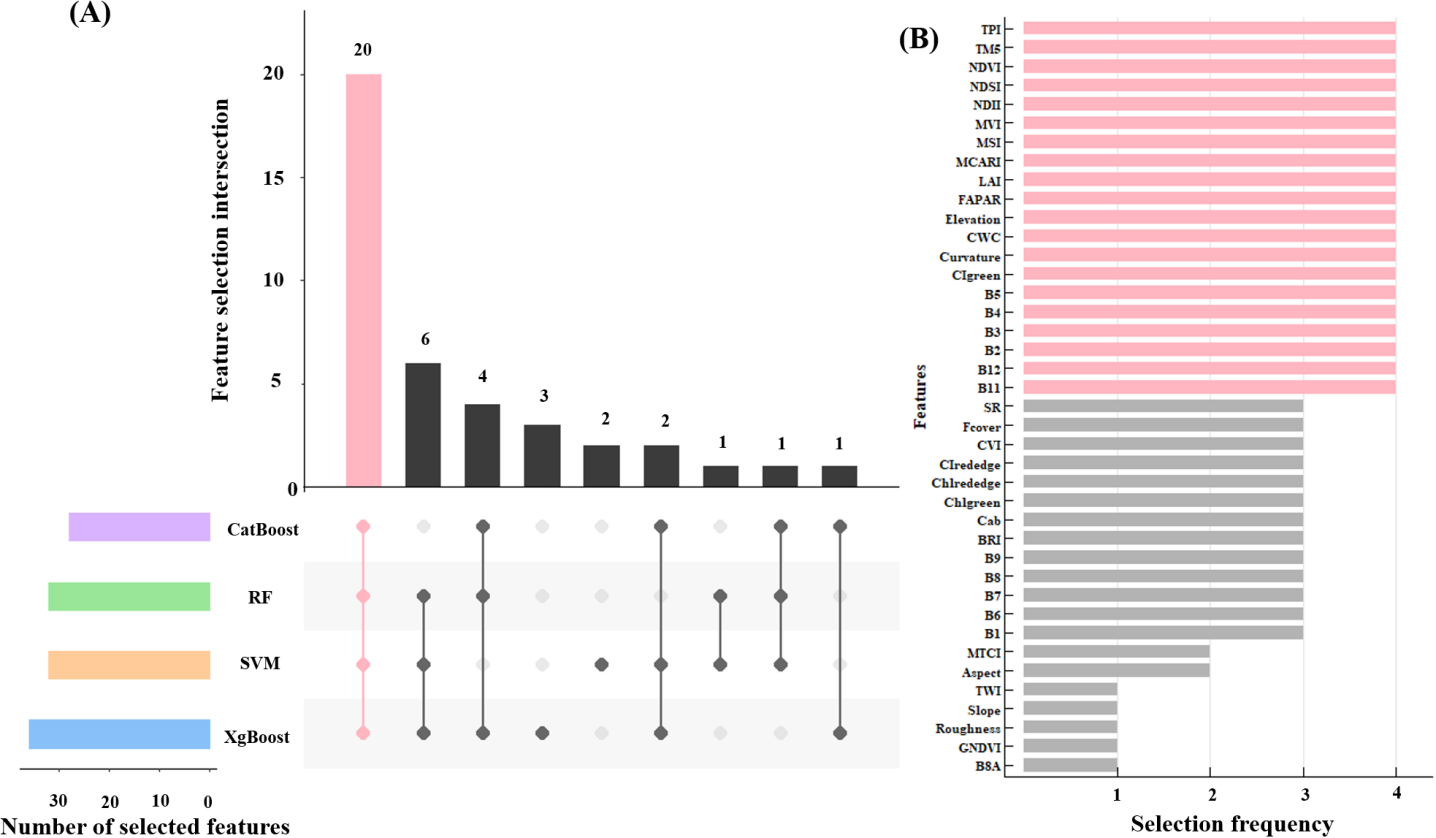
Feature selection results based on the RFECV **(A)**, and the 20 feature variables after selecting (the red bar graph) **(B)**.

In this study, Recursive Feature Elimination with Cross-Validation (RFECV), which was a robust feature selection method that intelligently selects features by recursively removing the least important features and evaluating model performance using cross-validation at each step, was employed to select 40 remote sensing indicators, resulting in the identification of 20 key variables that were common across four machine learning models: RF, XgBoost, CatBoost, and SVM (**Figure 4**). For these 20 remote sensing indicators, the pearson correlation coefficient was calculated to quantitatively assess the linear correlation between each feature variable and total forest AGB. Subsequently, each model was trained using 20 selected feature set, and feature importance analysis was conducted through 100 repetitions of 5-fold cross-validation.

#### 2.3.4 Cross-validation and model parameter tuning

**Figure 5:**
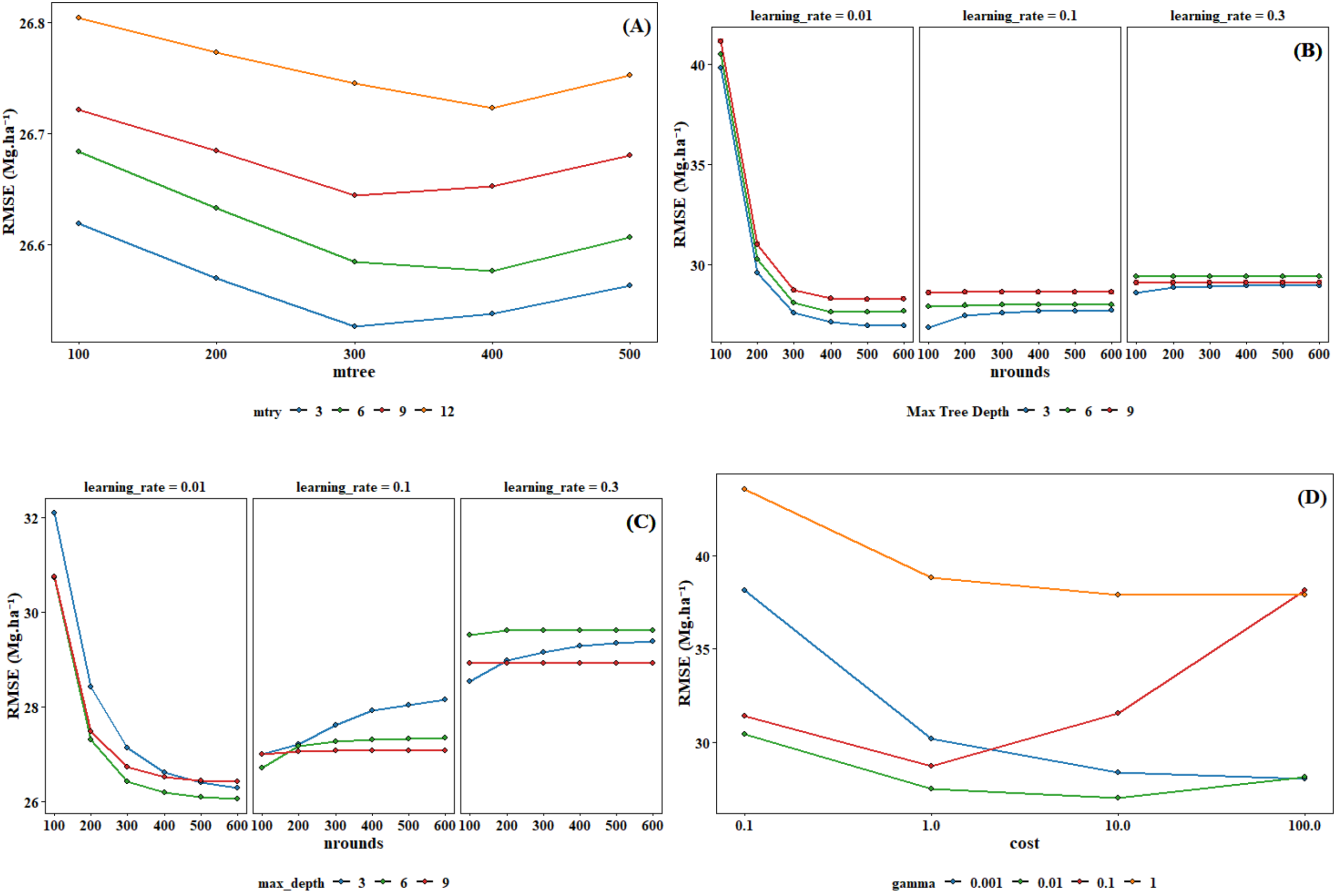
Parameter tuning for RF **(A)**, XgBoost **(B)**, CatBoost **(C)** and SVM **(D)**.

This study employed the grid search method combined with cross-validation for hyperparameter optimization. Grid search is an exhaustive search method, which searches for the optimal hyperparameters by traversing all possible combinations of hyperparameters. The hyperparameter ranges for different models in **Supplementary Table S2**. To enhance the reliability and generalizability of the model, a 5-fold cross-validation strategy was implemented during the hyperparameter tuning process. The original training dataset was randomly partitioned into five equal-sized subsets. In each iteration of cross-validation, four subsets were used as the training data to fit the model, while the remaining one subset was retained as the validation data for performance evaluation. This process was repeated five times, with each subset used exactly once as the validation set. The optimal hyperparameter combinations determined through the optimization process were presented in **Figure 5** and **Table 3**.

**Table 3:** Optimal training parameters for individual model.

Model	Parameter
RF	mtree=300
	mtry=6
XgBoost	nrounds=500
	max_depth=3
	learning_rate =0.01
CatBoost	nrounds=600
	max_depth=6
	learning_rate =0.01
SVM	Cost=10
	Gamma=0.01

#### 2.3.5 Development of stacked ensemble model

The stacked ensemble model integrates four distinct machine learning models—RF, XgBoost, CatBoost, and SVM—each offering unique strengths in capturing diverse data patterns. RF regression employs multiple decision trees on data subsamples and aggregates their predictions to enhance accuracy and reduce overfitting (Zhang et al., 2024). XgBoost is an efficient gradient boosting implementation that improves performance through regularization, sparsity-aware splitting, and parallel processing (Chen and Guestrin, 2016). CatBoost, also based on gradient boosting, excels at handling categorical features effectively while resisting overfitting and improving generalization (Luo et al., 2024). SVM completes the ensemble by identifying optimal hyperplanes for complex classification boundaries (Luo et al., 2024).

**Figure 6:**
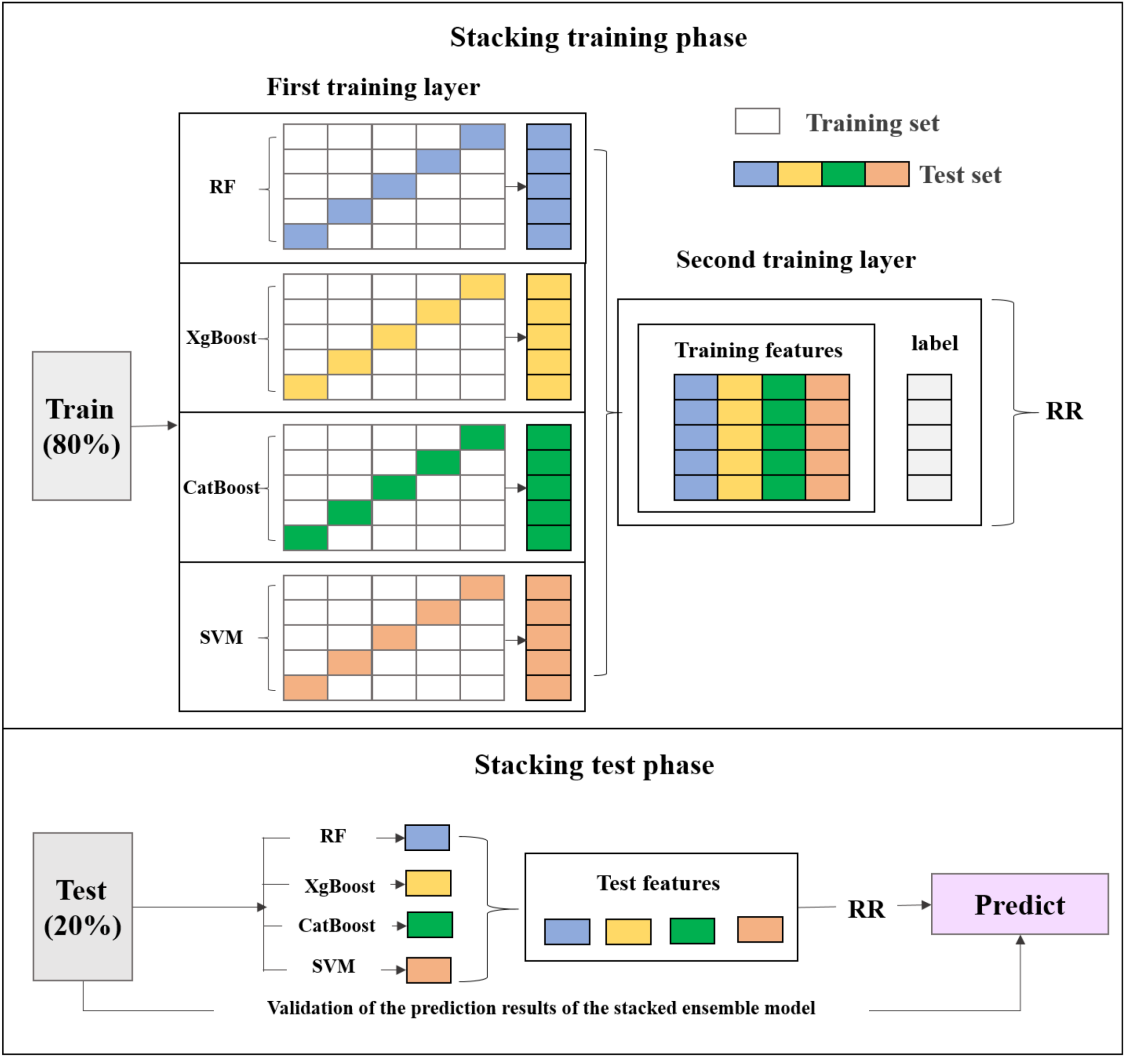
Framework of stacked ensemble model.

This study employs Ridge Regression (RR) as the meta-model in a stacked ensemble model. During the stacking process, the performance is evaluated using a sample-based cross-validation (CV) and external verification (EV). This study adopts 5-fold cross-validation that commonly used to test model robustness, where all site-based samples are randomly divided into five subsets. Each time, the model is trained on data from four subsets and tested on the remaining subset. In contrast, the EV experiment assesses the model’s generalization capability (i.e., true predictive performance) on a completely independent dataset that is not involved in any part of the model training process. In each iteration of the 5-fold cross-validation, the four base models are trained in parallel on the same training set and generate predictions on the test set. The predictions from the base models are used as new features, along with the target variable, to train the meta-model (RR). Subsequently, the external test values are input into the base models for training, and the results are fed into the trained stacked ensemble model for prediction. The final predictions are then validated (**Figure 6**).

#### 2.3.6 Model evaluation and uncertainty analysis

Model performance was evaluated using coefficient of determination (R^2^), mean absolute error (MAE) and root mean square error (RMSE) and root mean square error percentage (RMSE%). The calculation follows Equations 2–5.

(2) $$R^{2}\left(y,\;\hat{y}\right)=1-\frac{\sum_{i=1}^{n}{\left(y_{i}-\hat{y_{i}}\right)}^{2}}{\sum_{i=1}^{n}{\left(y_{i}-\bar{y}\right)}^{2}}$$

(3) $$MAE\left(y,\;\hat{y}\right)=\frac{1}{n}\overset{n}{\underset{i=1}{\sum }}\left|y_{i}-\hat{y_{i}}\right|$$

(4) $$RMSE\left(y,\;\hat{y}\right)=\sqrt{\frac{1}{n}\overset{n}{\underset{i=1}{\sum }}{\left(y_{i}-\hat{y_{i}}\right)}^{2}}$$

(5) $$RMSE\%(y,\;\hat{y})=\frac{\sqrt{\frac{1}{n}\sum_{i=1}^{n}{\left(y_{i}-\hat{y_{i}}\right)}^{2}}}{\bar{y}}*100\%$$

Where y and $\hat{y}$ represents actual and predicted values, respectively. $\bar{y}$ is the average actual values. n is the number of training datasets.

The uncertainty of the model was determined by the estimated values in cross validation, and the calculation follows Equation 6.

(6) $$Uncertainty=\sqrt{\frac{\sum_{j=1}^{n}{\left(p_{j}-\bar{p}\right)}^{2}}{n}}$$

**Figure 7:**
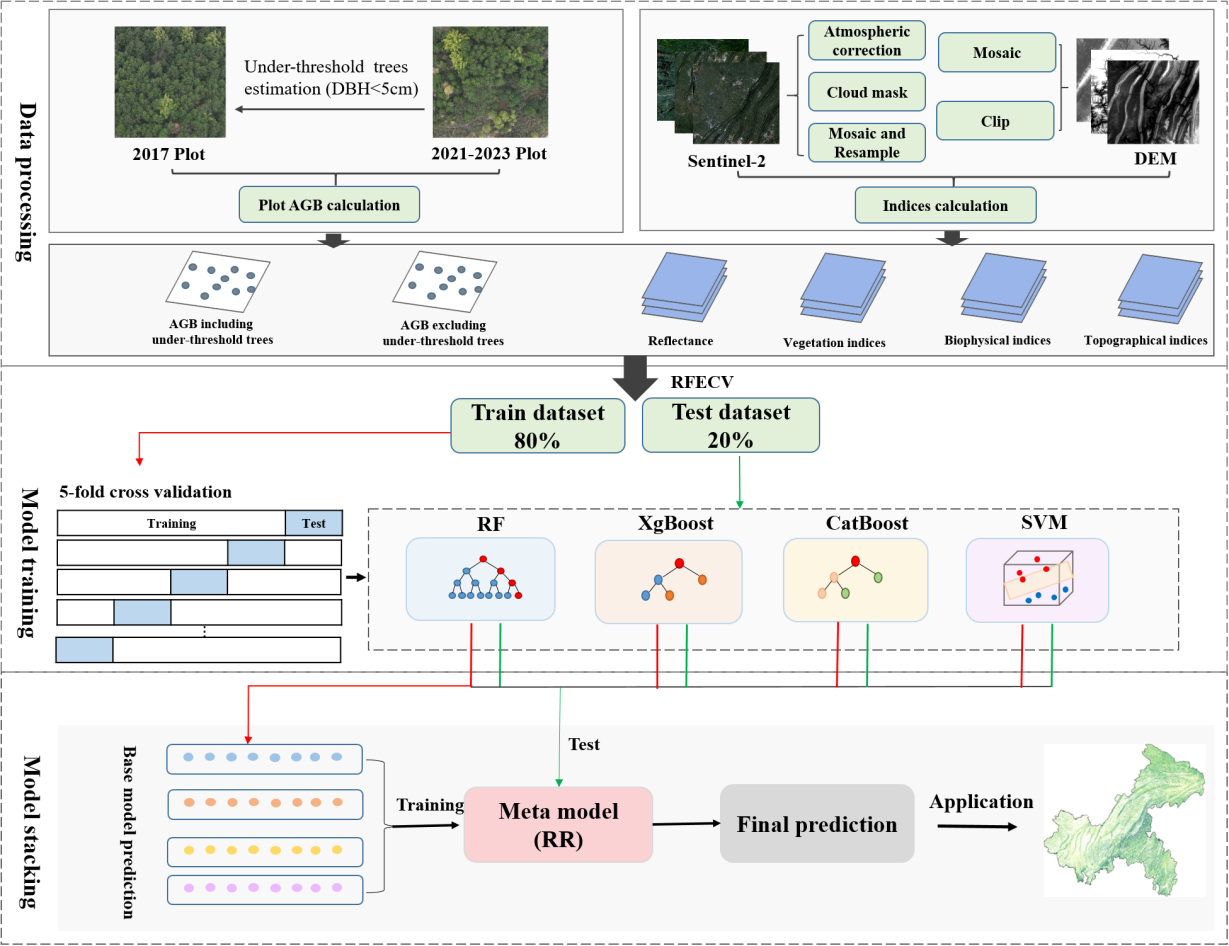
Workflows of AGB estimation based on field data, remote sensing images and machine learning.

Where n is 5 and $\;p_{1}$ is the predicted value of single-fold cross-validation. $\bar{p}$ is the average of the predicted values of the 5-fold cross-validation. The paper framework is shown in **Figure 7**.

## 3 Results

### 3.1 Estimating DBH of under-threshold trees

**Figure 8:**
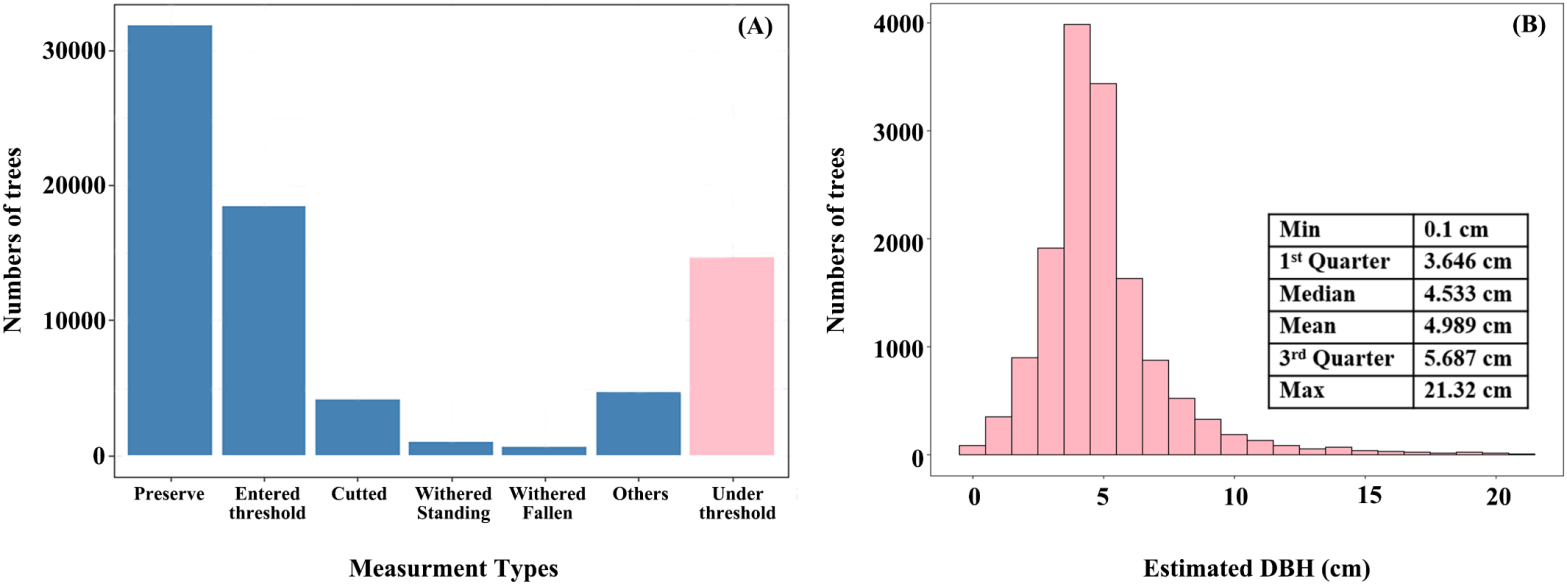
Distributionof tree numbers of different measure types **(A)** and estimated DBH of under-threshold trees **(B)**.

Under-threshold trees (DBH < 5 cm) in 2017 accounted for nearly 23% of all trees in plot data (14697 out of 60786 trees) (**Figure 8A**), and their estimated DBH distribution for under-threshold trees was consistent with NFI technique protocol, where most values fall around 5 cm (**Figure 8B**). The distribution mean and median were 4.983 cm and 4.533 cm, respectively, with interquartile ranging from 3.646 cm to 5.687 cm. Some outliers exceeding 10cm may result from measurement errors, such as boundary positioning errors between two adjacent NFI or operation errors in data collection.

### 3.2 Spatial distribution of plot-level AGB

**Figure 9:**
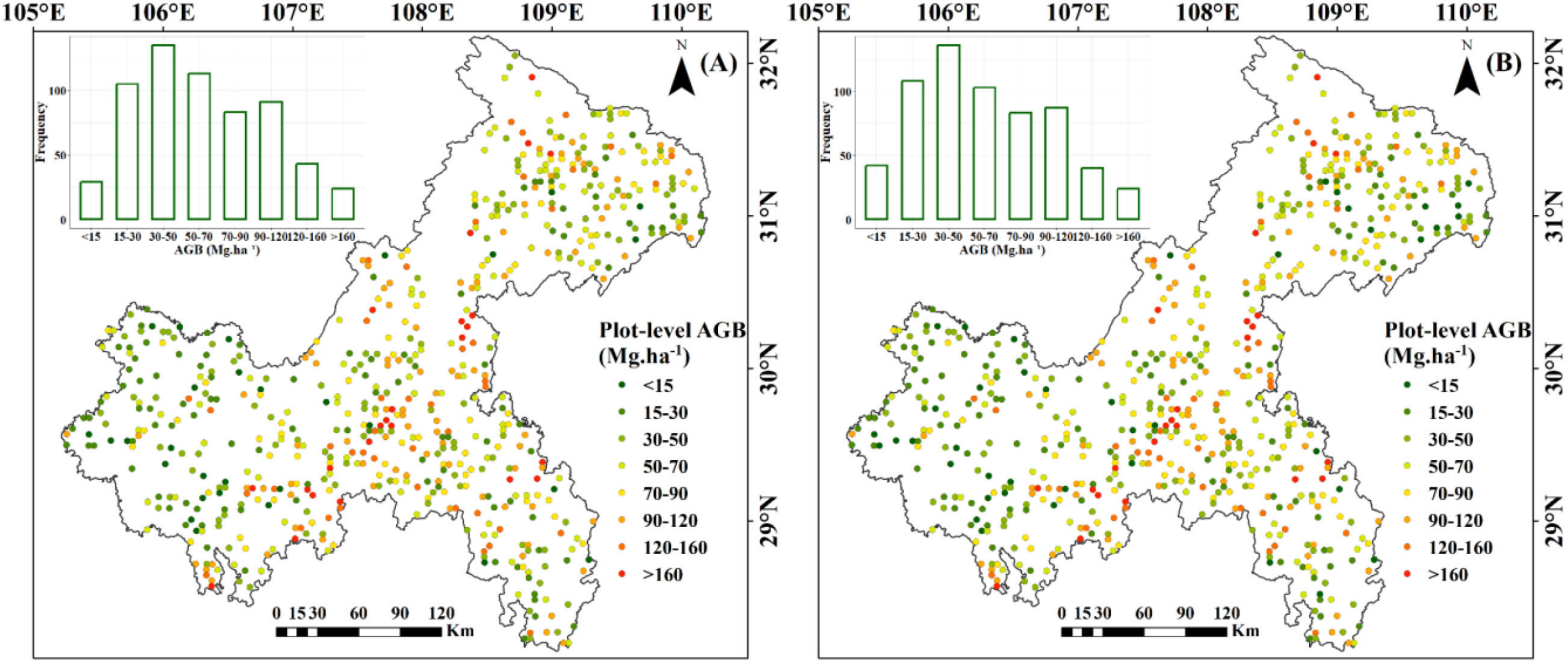
Spatial distribution of plot-level AGB: **(A)** Including under-threshold trees; **(B)** Excluding under-threshold trees.

**Figure 10:**
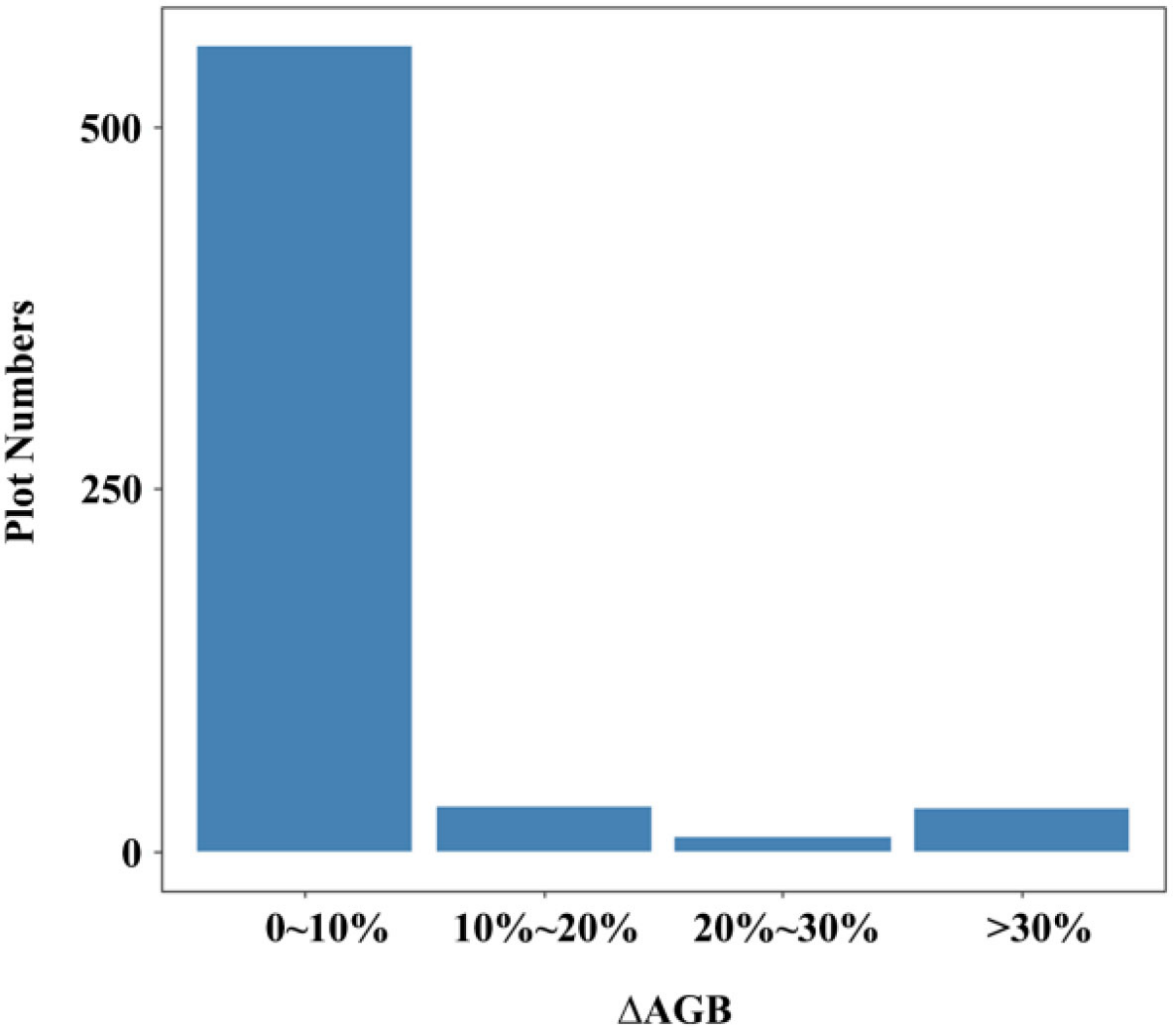
Distribution of changes in plot-level AGB after accounting for under-threshold trees.

The spatial distribution of the 623 NFI plots used in this study was presented in **Figure 9**. The plots were distributed relatively even across Chongqing to ensure representative spatial coverage. Plot-level AGB values calculated by allometric equations, including under-threshold trees, ranged from 5.15 to 329.81 Mg·ha^-1^, with an average of 66.63 Mg·ha^-1^. The majority of AGB across these plots fell between 33.38 and 90.48 Mg·ha^-1^ (**Table 4**). The median and mean plot-level AGB, excluding under-threshold trees, were relatively small, with values of 55.08 Mg·ha^-1^ and 64.48 Mg·ha^-1^. The overall range was from 0.62 Mg·ha^-1^ to 329.81 Mg·ha^-1^. Plot-level AGB values in western Chongqing were predominantly less than 50 Mg·ha-1, while plot-level AGB values exceeding 150 Mg·ha^-1^ were primarily concentrated along a northeast-southwest mountain ranges (**Figure 9**). When considering under-threshold trees, the increments of plot-level AGB values in more than 500 plots were within 10%, while the plot-level AGB values in the rest plots experienced increments exceeding 10% (**Figure 10**).

**Table 4:** Summary of plot-level AGB (Mg·ha^-1^).

Type	Min	1st Quarter	Median	Mean	3rd Quarter	Max
AGB of including under-threshold trees	5.15	33.38	57.43	66.63	90.48	329.81
AGB of excluding under-threshold trees	0.62	30.76	55.08	64.48	86.70	329.81

### 3.3 Features importance and relevance

**Figure 11:**
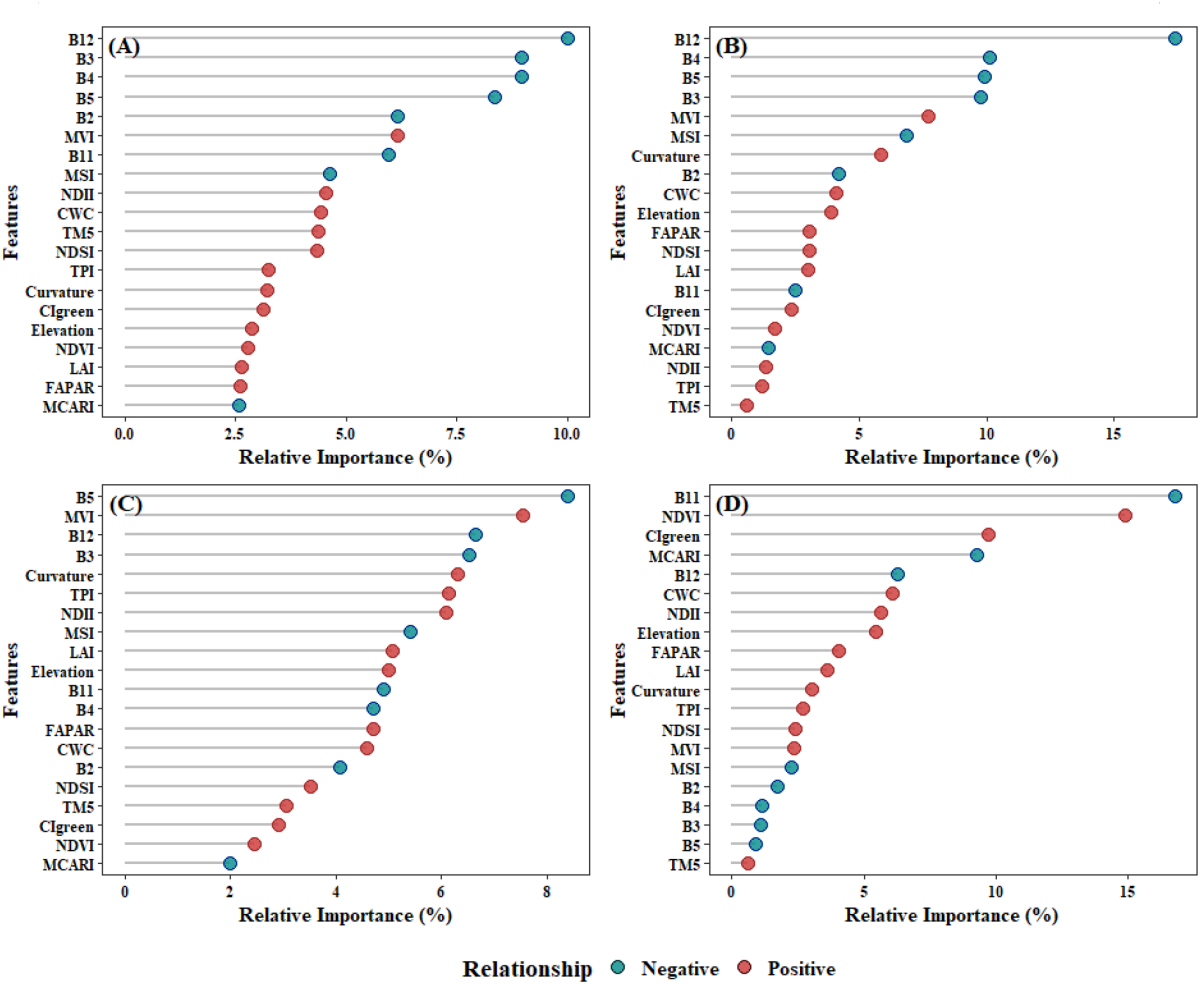
The correlation between features and AGB and the relative importance of features across different models: **(A)**RF; **(B)** XgBoost; **(C)**Catboost; **(D)** SVM.

In the RF model, the prediction of AGB primarily relied on spectral bands, with vegetation indices playing a secondary role (**Figure 11A**). Notably, B12, B4, B3, and B5 exhibited the highest relative importance at 12.1%, 9.5%, 8.9% and 7.7%, respectively, all showing significant negative correlations with AGB. Vegetation indices derived from spectral bands, such as MVI, MSI, and NDII, also demonstrated relatively high importance. In contrast, topographic features including Curvature and Elevation have relatively weaker importance.

The XgBoost model highlighted the critical importance of spectral bands in predicting AGB, with B12, B5, B3, and B4 identified as the most influential features, contributing 19.9%, 9.45%, 8.6%, and 8.5% to the model’s predictive power, respectively (**Figure 11B**). Among vegetation indices, MVI and MSI also played notable roles, accounting for 5.9% and 5.8% of the feature importance. Topographic indices, including Curvature, Elevation, significantly enhanced AGB prediction. Additionally, biophysical indices such as CWC demonstrated moderate importance, exhibiting positive feedback effects on AGB estimation.

In CatBoost model, spectral band and vegetation indices remain the most significant features (**Figure 11C**). Among these, B5 (7.1%) and MVI (6.8%) exhibited the highest relative importance, yet they demonstrated opposing feedback effects on AGB. These were followed by TPI in terms of feature contribution, while other topographic indices, such as Curvature and Elevation, showed moderate importance. In contrast, B12, NDII, and B3 were also identified as highly important features in the model.

In the SVM model, B11 (16.6%) and NDVI (15.2%) were the primary contributors, while vegetation indices such as CIgreen (9.5%) and MCARI (9.4%) were of secondary importance. The significance of topographic features remains moderate. Notably, features such as B2 (1.8%), B4 (1.1%), B3 (1.0%), and B5 (0.9%), which exhibited high importance in the other three models, showed relatively low importance in SVM (**Figure 11D**).

### 3.4 Model performances

**Figure 12:**
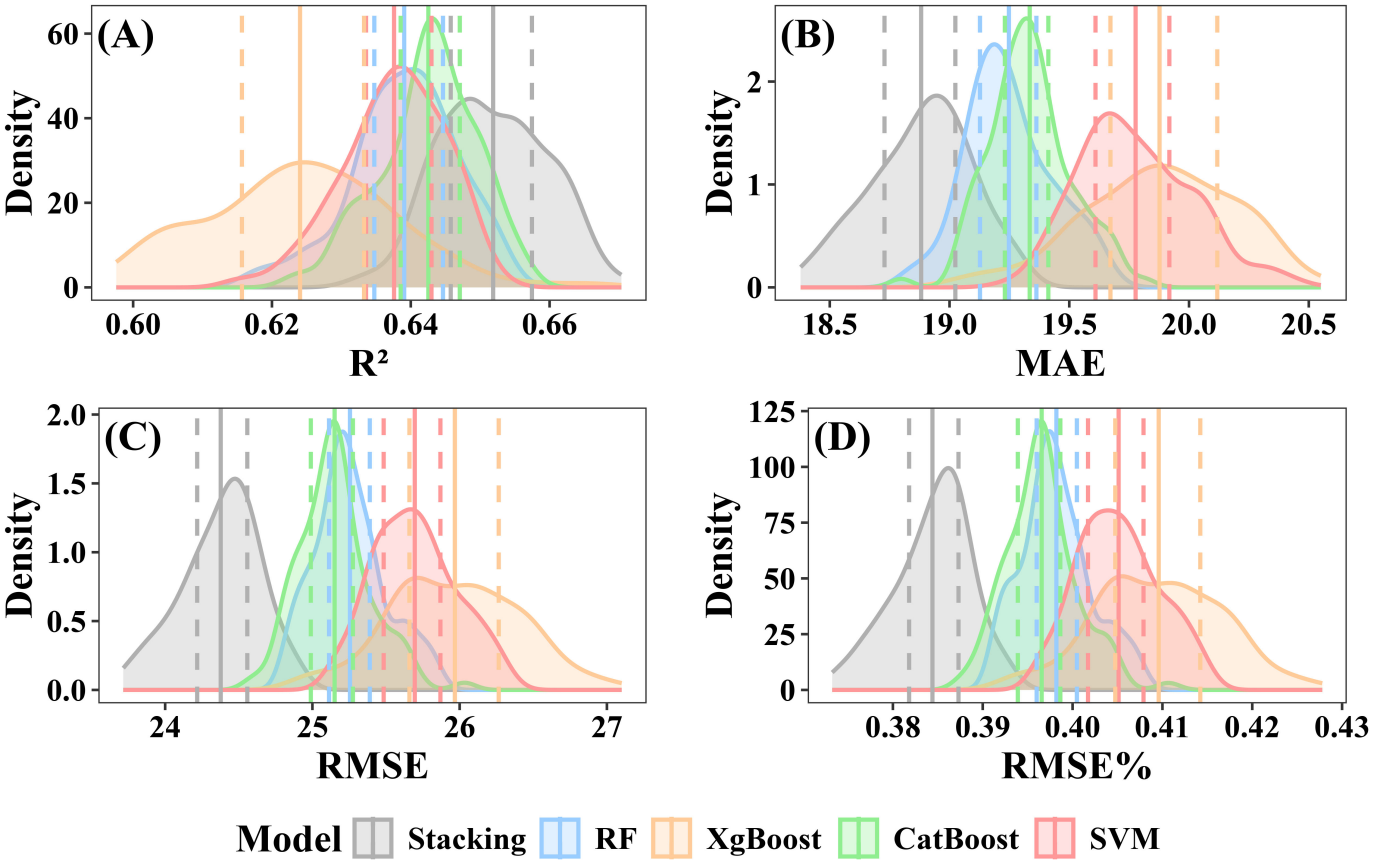
Performance evaluation of individual and stacked ensemble model including under-threshold trees. **(A)** R2; **(B)** MAE; **(C)** RMSE; **(D)** RMSE%.

The CV was used to evaluate the stability of the models. Both individual and stacked ensemble model demonstrated robust performance in estimating forest AGB. When including under-threshold trees, the CatBoost model demonstrated the highest predictive accuracy among all individual models, with a mean R² of 0.64 (interquartile range: 0.639–0.647) and a mean RMSE of 25.15 Mg·ha ^-^¹ (interquartile range: 24.99–25.28 Mg·ha ^-^¹) (**Figure 12C**). In contrast, XgBoost exhibited the lowest accuracy, with a mean R² of 0.62 (interquartile range: 0.616–0.633) and a mean RMSE of 25.97 Mg·ha ^-^¹ (interquartile range: 25.66–26.27 Mg·ha ^-^¹) (**Figure 12B**). The RF model showed intermediate performance, with a mean R² of 0.64 (interquartile range:0.635–0.645) and a mean RMSE of 25.26 Mg·ha ^-^¹ (interquartile range: 25.11–25.39 Mg·ha ^-^¹) (**Figure 12A**). SVM’s performance was slightly lower than that of RF, with a mean R² of 0.64 (interquartile range:0.634–0.643) and a mean RMSE of 25.70Mg·ha ^-^¹ (interquartile range: 25.48–25.87 Mg·ha ^-^¹) (**Figure 12D**). The stacked ensemble model slightly improved prediction accuracy, with a mean R² of 0.65 (interquartile range: 0.646–0.657) and an RMSE of 24.38 Mg·ha ^-^¹ (interquartile range: 24.22 to 24.56 Mg·ha ^-^¹) (**Figure 12E**).

**Figure 13:**
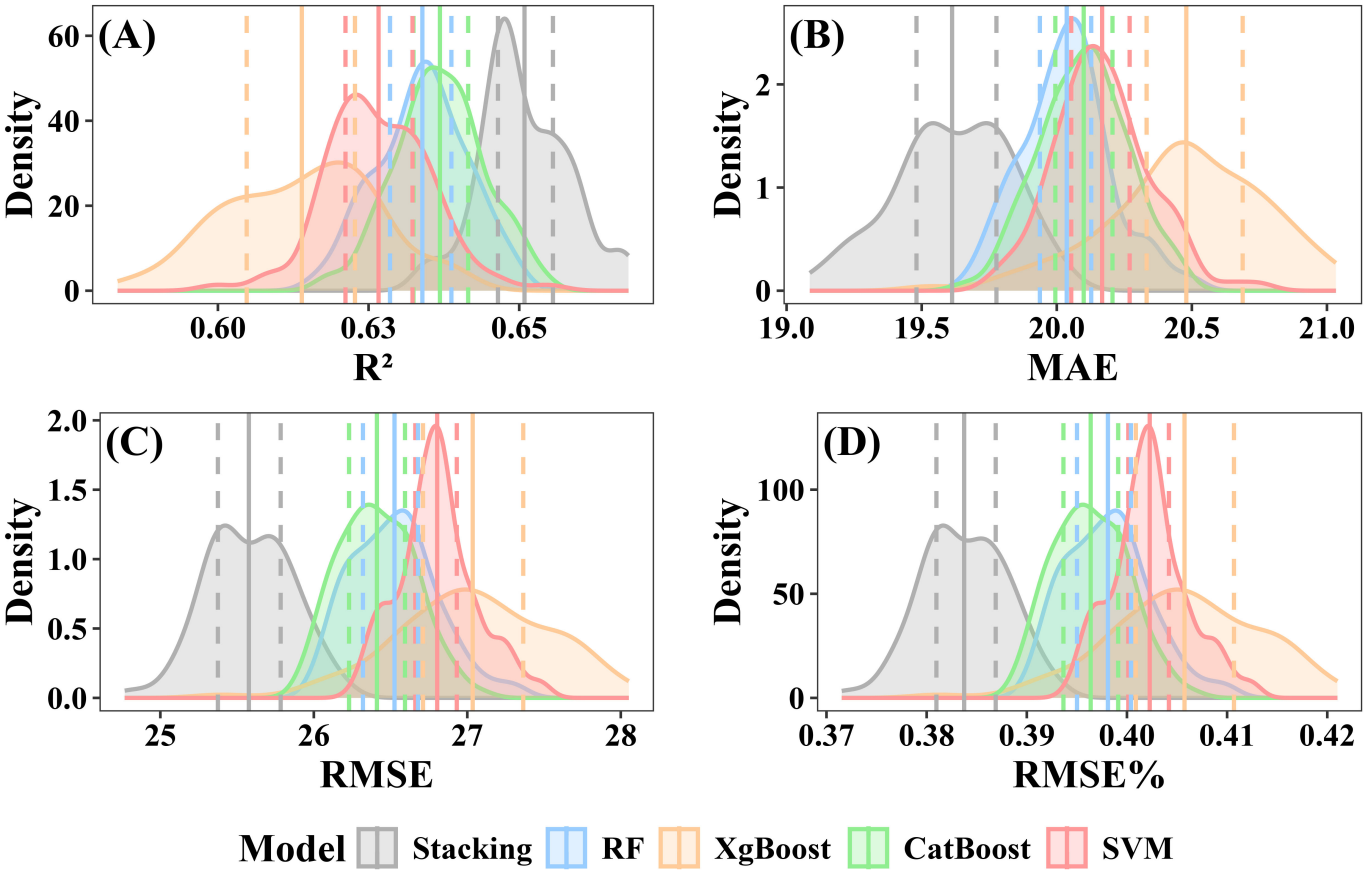
Performance evaluation of individual and stacked ensemble model excluding under-threshold trees. **(A)** R2; **(B)** MAE; **(C)** RMSE; **(D)** RMSE%.

When excluding under-threshold trees, the stacked ensemble model consistently outperformed individual models in prediction accuracy, achieving a mean R² of 0.65 (interquartile range: 0.646–0.656) and a mean RMSE of 25.58 Mg·ha ^-^¹ (interquartile range:25.38–25.78 Mg·ha ^-^¹) (**Figure 13E**). Among the individual models, CatBoost achieved a mean R² of 0.64 (interquartile range: 0.632–0.642) and a mean RMSE of 26.41 Mg·ha ^-^¹ (interquartile range: 26.23–26.59 Mg·ha ^-^¹) (**Figure 13C**). The RF model exhibited slightly lower accuracy, with a mean R² of 0.63 (interquartile range: 0.629–0.639) and a mean RMSE of 26.53 Mg·ha ^-^¹ (interquartile range: 26.32–26.68 Mg·ha ^-^¹) (**Figure 13A**). XgBoost displayed the weakest results, with a mean R² of 0.61 (interquartile range: 0.605–0.623) and a mean RMSE of 27.04 Mg·ha ^-^¹ (interquartile range: 26.71–27.36 Mg·ha ^-^¹) (**Figure 13B**). SVM mean R² and RMSE were respectively 0.62 (interquartile range: 0.621–0.632) and 26.80 Mg·ha ^-^¹(interquartile range: 26.66–26.93 Mg·ha ^-^¹) (**Figure 13D**).

**Figure 14:**
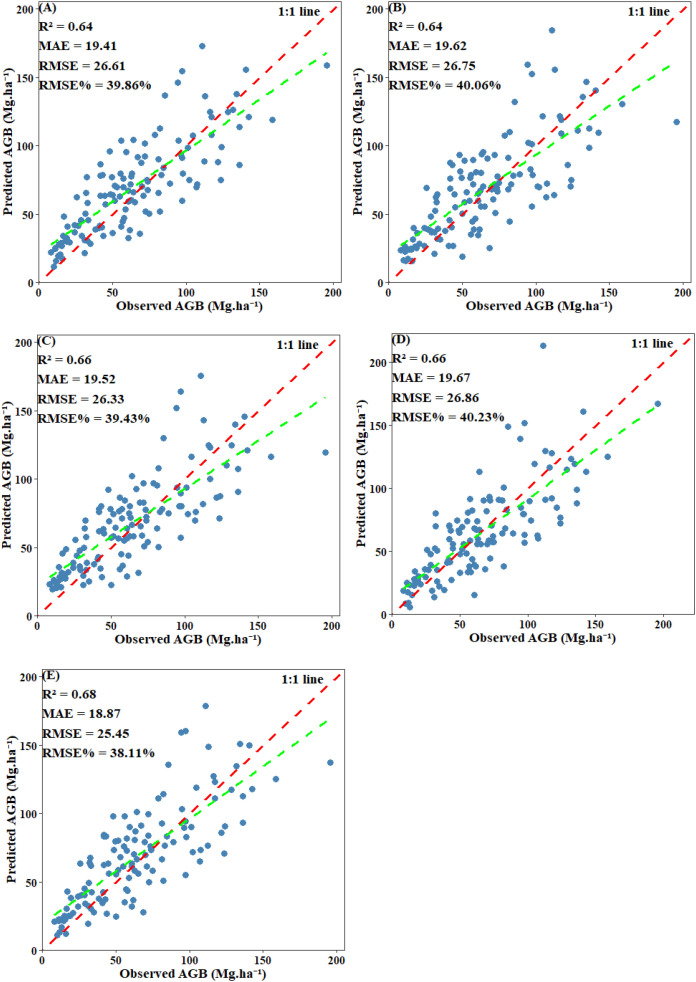
Scatter plot of predicted AGB and observed AGB with under-threshold trees included: **(A)** RF; **(B)** XgBoost; **(C)** CatBoost; **(D)** SVM; **(E)** Stacked ensemble model.

**Figure 15:**
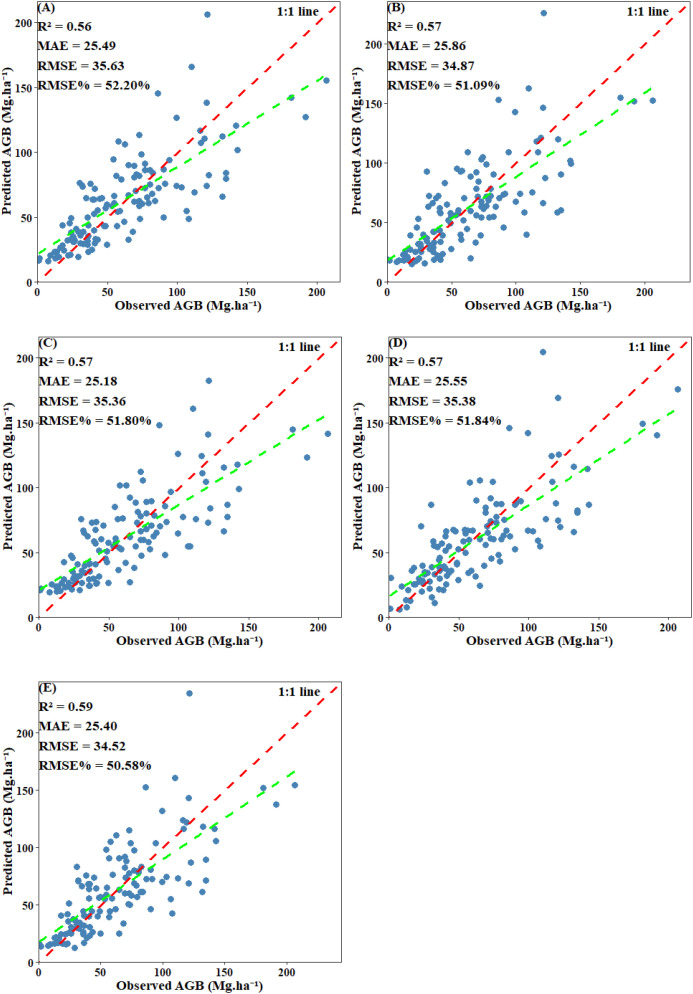
Scatter plot of predicted AGB and observed AGB with under-threshold trees excluded: **(A)** RF; **(B)** XgBoost; **(C)** CatBoost; **(D)** SVM; **(E)** Stacked ensemble model.

EV was used to evaluate the final performance of the model. Scatter plots comparing predicted values with plot-level calculated values revealed that all models displayed a systematic bias: they tended to overestimate AGB when values were below 30 Mg·ha ^-^¹ and underestimate AGB when it exceeded 125 Mg·ha ^-^¹. Among including below-threshold trees, the stacked ensemble model achieved the highest EV accuracy, with an R² of 0.68 and an RMSE of 25.45 Mg·ha ^-^¹. Both CatBoost and SVM also performed well, each attaining an R² of 0.66, with RMSE values of 26.33 Mg·ha ^-^¹ and 26.86 Mg·ha ^-^¹, respectively. RF and XgBoost also demonstrated competitive accuracy (R²: 0.64 and 0.64; RMSE: 26.61 Mg·ha ^-^¹ and 26.75 Mg·ha ^-^¹) (**Figure 14**). After excluding under-threshold trees, EV accuracy declined significantly. The R² values of RF, CatBoost, and SVM fell within the range of 0.56–0.57, and all RMSE values exceeded 35.36 Mg·ha ^-^¹. The stacked ensemble model somewhat improved predictive performance and reduced discrepancies among the base models (R²=0.59, RMSE=34.52 Mg·ha ^-^¹) (**Figure 15**). It can be concluded that although AGB predictions under both scenarios reached similar stability during model training, the inclusion of under-threshold trees substantially enhances the EV accuracy of the predictions.

### 3.5 Spatially forest AGB map and uncertainty analysis

**Figure 16:**
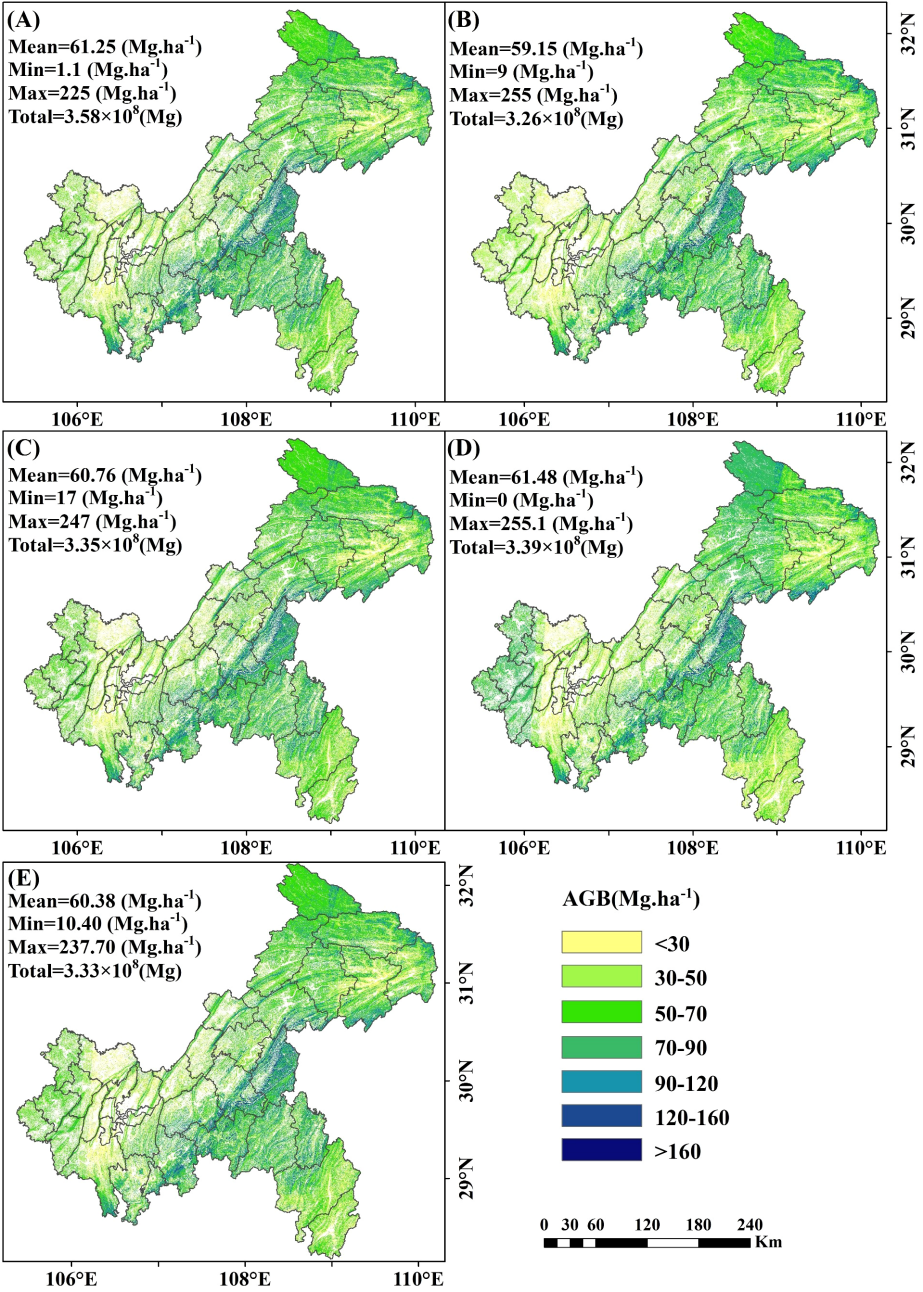
Spatial map of AGB including under-threshold trees at a 10 m resolution. **(A)** RF; **(B)** XgBoost; **(C)** CatBoost; **(D)** SVM; and **(E)** Stacked ensemble model.

The 10m resolution map revealed clear distinct patterns in AGB estimation with and without under-threshold trees. When including under-threshold trees, the average AGB values for the RF, XgBoost, CatBoost, SVM and stacked ensemble models were 61.25 Mg·ha ^-^¹,59.15 Mg·ha ^-^¹, 60.76 Mg·ha ^-^¹, 61.48 Mg·ha ^-^¹ and 60.38 Mg·ha ^-^¹, respectively, with corresponding total AGB values of 3.58×10^8^ Mg, 3.26×10^8^ Mg, 3.35×10^8^ Mg, 3.39×10^8^ Mg, and 3.33×10^8^ Mg (**Figure 16A-E**). When excluding under-threshold trees, the average AGB values for RF, XgBoost, CatBoost, SVM and the stacked ensemble model decreased to59.29 Mg·ha ^-^¹, 57.08 Mg·ha ^-^¹, 58.61 Mg·ha ^-^¹, 61.11 Mg·ha ^-^¹, and 58.70 Mg·ha ^-^¹, respectively, with total AGB values of 3.27×10^8^ Mg, 3.15×10^8^ Mg, 3.23×10^8^ Mg, 3.37×10^8^ Mg, and 3.24×10^8^ Mg (**Supplementary Figure S1A-E**). The spatial distribution patterns of AGB maps extrapolated from different model implementations (in/ex-clude under-threshold trees data) were similar: lower AGB values were found in the western and northeastern regions of Chongqing, while higher AGB concentrations were mainly located in the southeastern area.

**Figure 17:**
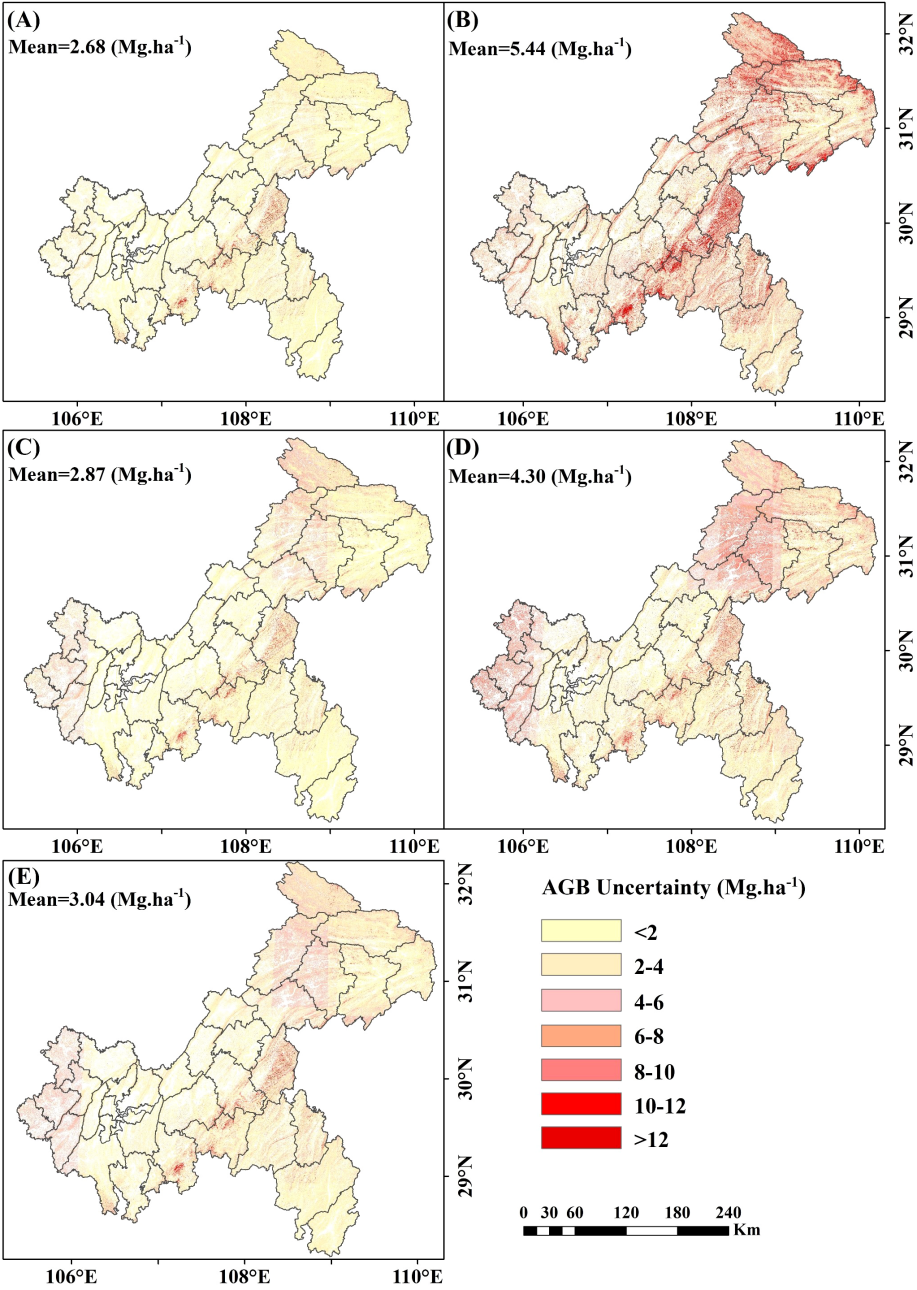
Spatial distribution of uncertainty of AGB including under-threshold trees. **(A)** RF; **(B)** XgBoost; **(C)** CatBoost; **(D)** SVM; and **(E)** Stacked ensemble model.

Uncertainty analysis revealed that the uncertainty associated with including under-threshold trees was lower than that when excluding them. When under-threshold trees were included, the average uncertainties for RF, XgBoost, CatBoost, and SVM were 2.68 Mg·ha ^-^¹, 5.44 Mg·ha ^-^¹, 2.87 Mg·ha ^-^¹, and 4.30 Mg·ha ^-^¹, respectively. The stacked ensemble model further reduced uncertainty to 3.04 Mg·ha ^-^¹ (**Figure 17A-E**). In the scenario where under-threshold trees were excluded, the average uncertainties for RF, XgBoost, CatBoost, SVM, and the stacked ensemble model were 2.77 Mg·ha ^-^¹, 5.47 Mg·ha ^-^¹, 2.94 Mg·ha ^-^¹, 4.37 Mg·ha ^-^¹, and 3.11 Mg·ha ^-^¹, respectively (**Supplementary Figure S2A-E**). Areas with higher uncertainty were primarily distributed in northeastern Chongqing and mountainous regions, while regions with lower uncertainty were mainly concentrated in central and western Chongqing. Additionally, the uncertainties of the RF and CatBoost models were significantly lower than those of XgBoost and SVM, with the stacked ensemble model exhibiting intermediate levels of uncertainty.

## 4 Discussion

### 4.1 Under-threshold trees should be considered in AGB estimation

According to the technical protocol of the NFI, all trees (including economic trees), bamboo (including bamboo in non-bamboo forests), and tree-like shrubs (excluding shrub-like tree species) in permanent plots should be measured if their DBH reaches 5 cm. Previous studies which utilized NFI data for forest AGB estimation generally did not focus on the impact of trees with DBH less than 5 cm (Zhu et al., 2020; Qian et al., 2021; Li et al., 2022; Zhang et al., 2023), as researchers generally assumed that the biomass contribution of these trees was negligible. However, we compared two consecutive NFI datasets in this study and employed a developed DBH estimation method to estimate the number of trees with DBH less than 5 cm in the earlier measurement. The results indicated that under-threshold trees account for a significant proportion (23%) of the total number of trees measured in 2017, with some plots having AGB variations exceeding 10% (**Figure 9** and **Figure 10**). Although the AGB change of tree plots under threshold value was small, in these plots, the under-threshold trees were primarily broadleaf trees, including *Cupressus funebris*, *Cyclobalanopsis glauca*, *Pinus massoniana*, *Cunninghamia lanceolata*, and *quercus* spp. The studies conducted by Yang et al. (2012) and Wen et al. (2015) found that in southern and southwestern China, new wild tree species began to emerge in the forests of *Pinus massoniana*, *Cyclobalanopsis glauca* and other broad-leaves forest after the middle-aged forest stage, and community succession existed in over-mature forests to a certain extent. Based on the 2017 forest resource survey data from our study, middle-aged forests, near-mature forests and over-mature forests collectively occupied a significant proportion (42%) of the forest sub-compartments. In addition, excluding under-threshold trees in AGB estimation resulted in noticeably lower R² values and higher RMSE values across all models, indicating a significant decline in prediction accuracy (**Figure 14** and **Figure 15**). These results underscored the non-negligible importance of under-threshold trees in AGB estimation.

In the forest AGB predicted by including under-threshold trees, higher values were primarily concentrated in the Daba, Wulingshan, and Dalou mountain regions. Firstly, these three areas feature high altitudes and inherently possess high forest coverage, leading to elevated forest AGB. Secondly, the 2015 “National Ecological Function Zoning” designated these regions as important areas for water conservation and biodiversity protection, strengthening the protection of existing nature reserves and the management of natural forests. For damaged ecosystems, efforts have been made to adhere to natural restoration, rejuvenate the tree, shrub, and grass vegetation system of evergreen broad-leaved forests, optimize the structure of forest ecosystems, continue implementing the Grain for Green Program and grassland restoration projects, as well as rocky desertification control projects, further enhancing forest coverage. Thirdly, Chongqing has adopted management measures such as establishing multiple nature reserves and forest parks to reduce the impact of human activities, which contributes to increased forest AGB. Areas with lower forest AGB were mainly distributed in the western part of Chongqing, primarily due to frequent human activities and low forest coverage.

### 4.2 Importance of features on AGB estimation

Feature variables were pivotal in forest AGB estimation. In the study, spectral bands emerged as the most critical features across all models, with B3, B5, and B12 demonstrating consistently high importance, corroborating findings by Wai et al. (2022) (**Figure 11**). B5, a red-edge band, exhibited strong capabilities in detecting key vegetation physiological parameters, including chlorophyll content and canopy architecture, which were essential for accurate AGB estimation (Zhang et al., 2023). In contrast, other red-edge bands (B6, B7) have shown greater significance in previous studies (Yang et al., 2012). B3, located at the chlorophyll reflection peak (500–600 nm), was highly sensitive to vegetation “greenness” and effectively reflected physiological states such as chlorophyll content and photosynthetic activity. Similarly, B2 and B4, also within the visible spectrum, were notably important in RF and XgBoost models (Wen et al., 2015). The effectiveness of shortwave infrared (SWIR) bands, particularly B12, in forest AGB estimation had been well-documented. B12 was sensitive to vegetation water content. As biomass and vegetation coverage increased, reflectance absorption by vegetation or water reduced reflectance, whereas low biomass areas exhibited higher reflectance, explaining the negative correlation between B12 and AGB (Wai et al., 2022). These findings underscored the high sensitivity of SWIR, visible, and red-edge bands to biomass, highlighting their critical role in biomass assessment.

The importance of vegetation indices varied across models. MVI was consistently significant across all three models, primarily reflecting forest canopy characteristics that contribute to biomass accumulation. Indices such as MSI and NDII also showed notable importance. MSI typically exhibited a negative feedback effect on AGB, driven by vegetation’s dependence on water and the impact of water stress on growth and physiological processes. NDII and SR primarily indicated changes in foliar chlorophyll and carotenoid content, reflecting photosynthetic activity, becoming key parameters for AGB remote sensing retrieval (Richardson et al., 2002; Merzlyak et al., 2003; Main et al., 2011).

Topographic features, including TPI, Curvature, and Elevation, were strong predictors of forest AGB, particularly in the CatBoost model. Chongqing’s mountainous terrain, characterized by diverse geomorphological features, influences sunlight exposure and water retention, thereby affecting vegetation growth (Wai et al., 2022). TPI and curvature effectively captured these land surface variations. Elevation and aspect also showed significant importance, consistent with previous research (Chen et al., 2019b; Wang et al., 2021). In mountainous regions, variations in elevation and aspect impact moisture levels, temperature, and species richness, ultimately influencing vegetation biomass (Shen et al., 2014; Cong et al., 2019).

### 4.3 Model performance in AGB estimation

Among the individual employed models evaluated, CatBoost demonstrated the best performance, achieving an R² of 0.66, followed by RF and XgBoost, while SVM yielded comparatively lower results (**Figure 9**). Previous studies have indicated that tree-based machine learning methods were particularly well-suited for ecological remote sensing research (Belgiu and Dragut, 2016). The RF, CatBoost and XgBoost models evaluated in this study were all ensemble methods based on decision trees, and the AGB maps generated by them have a high degree of spatial consistency. Compared with RF and XgBoost, CatBoost was a better choice for estimating AGB due to its advanced design and functions. CatBoost employed an ordered boosting mechanism that reduced the risk of overfitting and minimized the impact of noisy data by processing training examples in a specific order. Given that NFI data often contain inherent noise and variability, CatBoost’s robust handling of categorical features and superior generalization capabilities, makes it particularly well-suited for accurately estimating AGB in complex and noisy datasets. Based on individual models, a stacked ensemble model for AGB estimation using RR as the meta-model was developed. The stacked ensemble model significantly improved prediction accuracy, generalization capability, and robustness, achieving an R² of 0.68 and highlighting its superior performance (**Figure 14**). The selection of RR as the meta-model was based on the following considerations. Compared to alternative meta-models such as LM, KNN, and entropy weighting, RR exhibited superior and highly consistent performance in both CV and EV, with no evidence of overfitting (**Supplementary Table S3**). Furthermore, since the base models—RF, XgBoost, and CatBoost—were all tree-based models, their predictions were prone to high correlation (collinearity). RR effectively mitigated collinearity through L2 regularization, yielding more stable and reliable coefficient estimates, although RR’s CV accuracy was slightly lower than that of LM. KNN achieved the highest CV performance, the test performance of KNN declined markedly—indicating overfitting and disqualifying it as a suitable meta-model. The entropy weight method performed similarly to LM and RR on CV, though marginally worse. However, this study found that although the stacked ensemble model demonstrated the highest performance and stronger generalization capability, its uncertainty was greater than that of RF and CatBoost. The primary reason for this is that the uncertainty of the stacked ensemble model is influenced not only by factors such as 5-fold cross-validation but also by performance variations among the base models. Therefore, future research should focus on developing stacked ensemble models that achieve high performance while maintaining low uncertainty. Additionally, the scarcity of field-measured data in high-altitude areas where are usually heavily vegetated resulted in insufficient training, leading to higher uncertainty across all models in these regions. This study also evaluated LM, ANN, and RR as base models and found their performance substantially lower than that of the tree-based ensemble (**Supplementary Table S4**). These results underscore the strong potential of tree-based machine learning models in AGB estimation.

**Figure 18:**
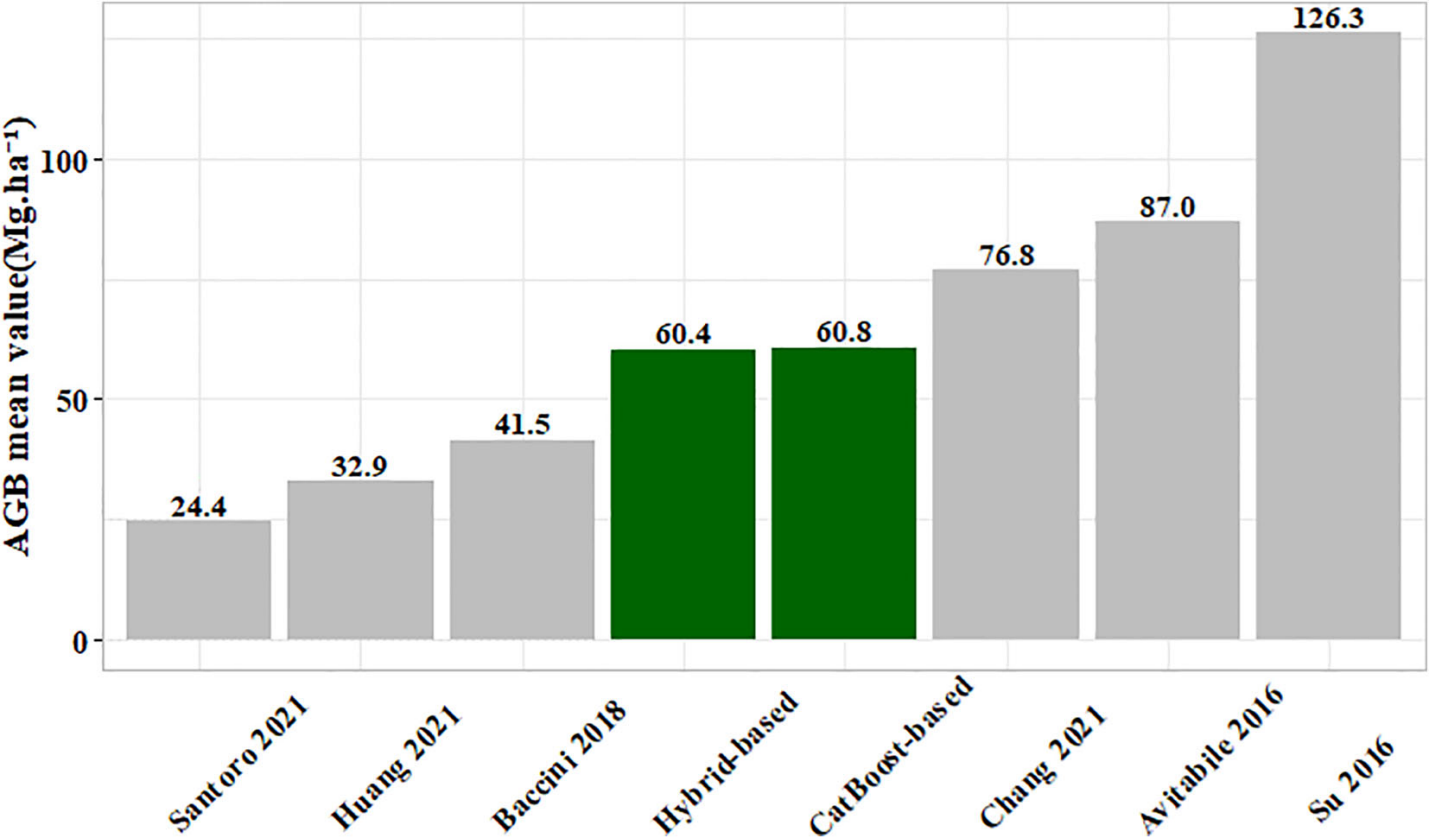
Comparison of present AGB map (including under-threshold trees) and different published AGB products.

To validate the generated 10 m spatial resolution forest AGB map, we compared it (including under-threshold trees) with existing AGB products (**Figure 18**). Our results aligned closely with those of Chang et al. (2021) in six studies (Avitabile et al., 2016; Su et al., 2016; Baccini et al., 2018; Huang et al., 2021; Santoro and Cartus, 2021). The mixed model RMSE in Chang’s study ranged between 24.3 Mg·ha ^-^¹ and 29.6 Mg·ha ^-^¹, while the stacked ensemble model RMSE in this study was 25.45 Mg·ha ^-^¹, indicating comparable model accuracy (**Figure 14E**). The reason for the differences might be the variations in the base model and the stacking method used. The model exhibited a slight overestimation at low AGB levels (<30 Mg·ha ^-^¹) but a significant underestimation at high AGB levels (>125 Mg·ha ^-^¹), consistent with documented saturation thresholds in AGB estimation that vary depending on remote sensing data, modeling approaches, and forest structure (Chen et al., 2018; Qian et al., 2021; Wai et al., 2022). First, remote sensing data limitations contributed to these errors. In low AGB areas, the dense canopy structure of small trees obscures thinner trunks (smaller DBH). The spatial resolution of Sentinel-2 (10–20 m) primarily captures spectral characteristics of leaves to estimate AGB, failing to adequately represent trunk structures, which leads to overestimation. Conversely, spectral saturation in high-biomass regions (particularly dense forests) reduced the ability of sensor to discriminate subtle vegetation differences. Although red-edge band of Sentinel-2 partially mitigated saturation, improper band combinations or model selection could still result in underestimation at high AGB levels. Second, model training limitations introduced additional biases. The scarcity of high-biomass samples in the training dataset led to insufficient learning of extreme values, causing the model to regress toward the mean and underestimate high AGB. Meanwhile, low AGB may be overestimated due to noise or mixed-pixel effects (e.g., soil background interference). Therefore, Addressing AGB saturation remained a significant challenge in remote sensing (Qian et al., 2021). Potential improvements to mitigate AGB underestimation include leveraging hyperspectral imagery and LiDAR data to construct three-dimensional forest models, as well as integrating climate and environmental data to enhance biomass estimation accuracy (Feng et al., 2024). Additionally, exploring novel methods such as parametric decomposition and clustering to characterize horizontal and vertical forest structure details could provide alternative approaches.

Computational performance considerations: For the AGB prediction using stacked ensemble models in this study, the computing device must have a minimum of 64 GB RAM and over 500 GB storage memory. The runtime for a single model under a specific scenario exceeds 24 hours. Therefore, it was recommended to implement this method on high-performance computers supporting parallel processing capabilities.

## 5 Conclusions

In this study, we developed a DBH estimation method using NFI data to measure DBH of under-threshold trees, integrated remote sensing imagery and topographic data to compare the performances of individual and stacked ensemble model between scenarios including and excluding under-threshold trees, and ultimately generated a 10m resolution forest AGB map for Chongqing.

The developed method for estimating the DBH of under-threshold trees demonstrated high accuracy (R²=0.93, RMSE=1.46 cm). Given that under-threshold trees constituted 23% of the total tree population, their exclusion significantly compromised the accuracy of AGB prediction. Consequently, in forest AGB remote sensing inversion studies utilizing NFI data, the calibration of trees with DBH < 5 cm is crucial to minimize deviation and improve prediction accuracy.

Spectral bands serve as the predominant features for AGB prediction across all models, while vegetation and topographic indices exhibited significant variations in their importance among different models. Consequently, the selection of distinct feature variables tailored to specific models contributes to enhanced prediction accuracy.

The stacked ensemble model demonstrated superior performance compared to individual models. Although all four individual models achieved R² values between 0.64 and 0.66 (including the under-threshold trees), the stacked ensemble model effectively reduced inter-model variability and improved prediction accuracy (R²=0.68), which was notably higher than the value obtained when excluding under-threshold trees (R²=0.59) These findings established a foundation for exploring the potential applications of hybrid machine learning approaches in forest AGB estimation.

## Data Availability

The original contributions presented in the study are included in the article/**Supplementary Material**, further inquiries can be directed to the corresponding author/s.
