# Supplementary material for: Estimation of forest above-ground biomass based on stacked ensemble model in Chongqing, China
Source: Front Plant Sci. 2025 Nov 7;16:1657170. doi: 10.3389/fpls.2025.1657170 (PMC12634669; doi:10.3389/fpls.2025.1657170)
Supplement: Supplementary file 1 [file DataSheet1.docx]

Supplementary Material

# Supplementary Figures and Tables

## Supplementary Figures


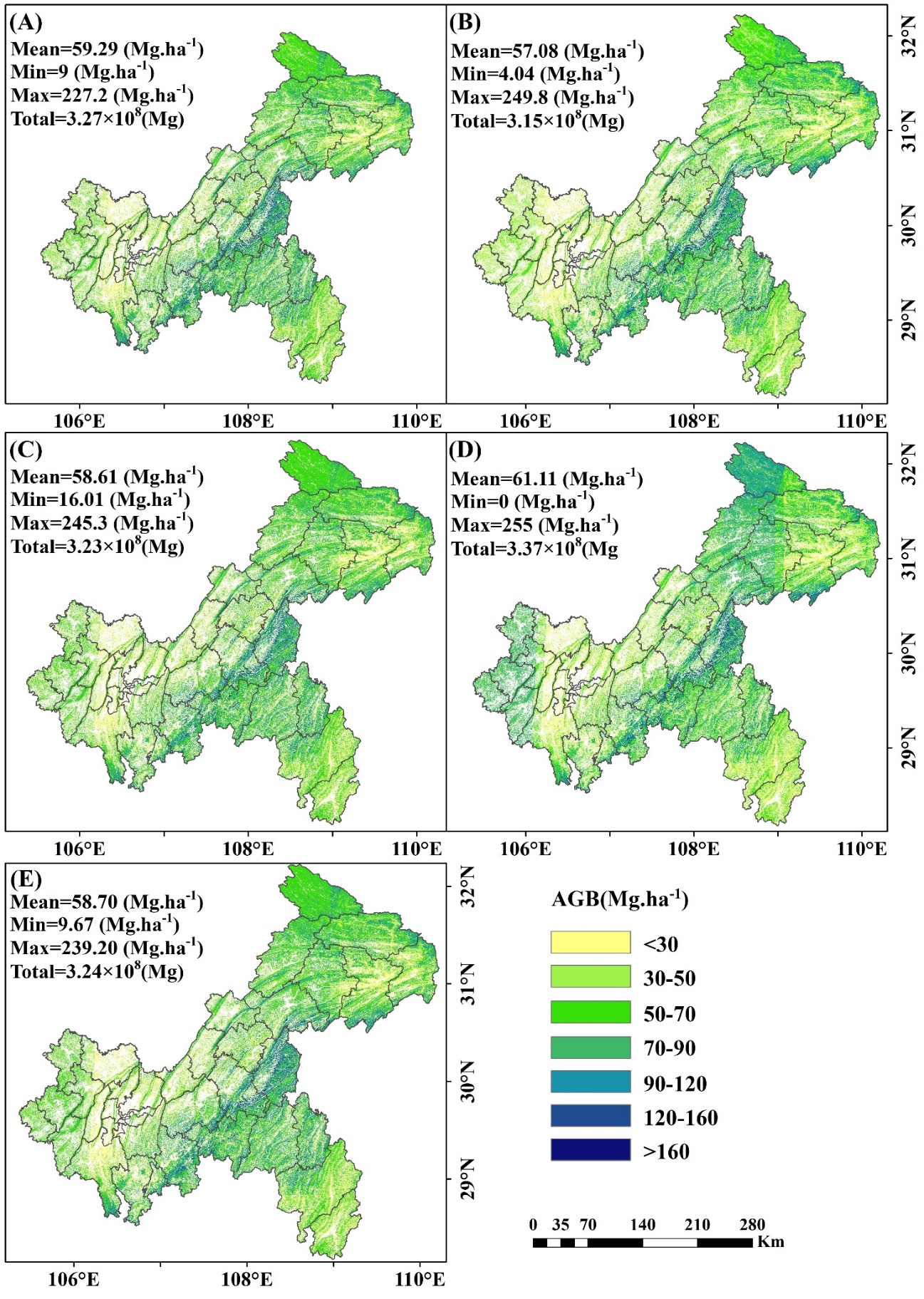


**Supplementary Figure S1.** Spatial map of AGB excluding under-threshold trees at a 10 m resolution. (A) RF; (B) XgBoost; (C) CatBoost; (D) SVM; and (E) Stacked ensemble model.


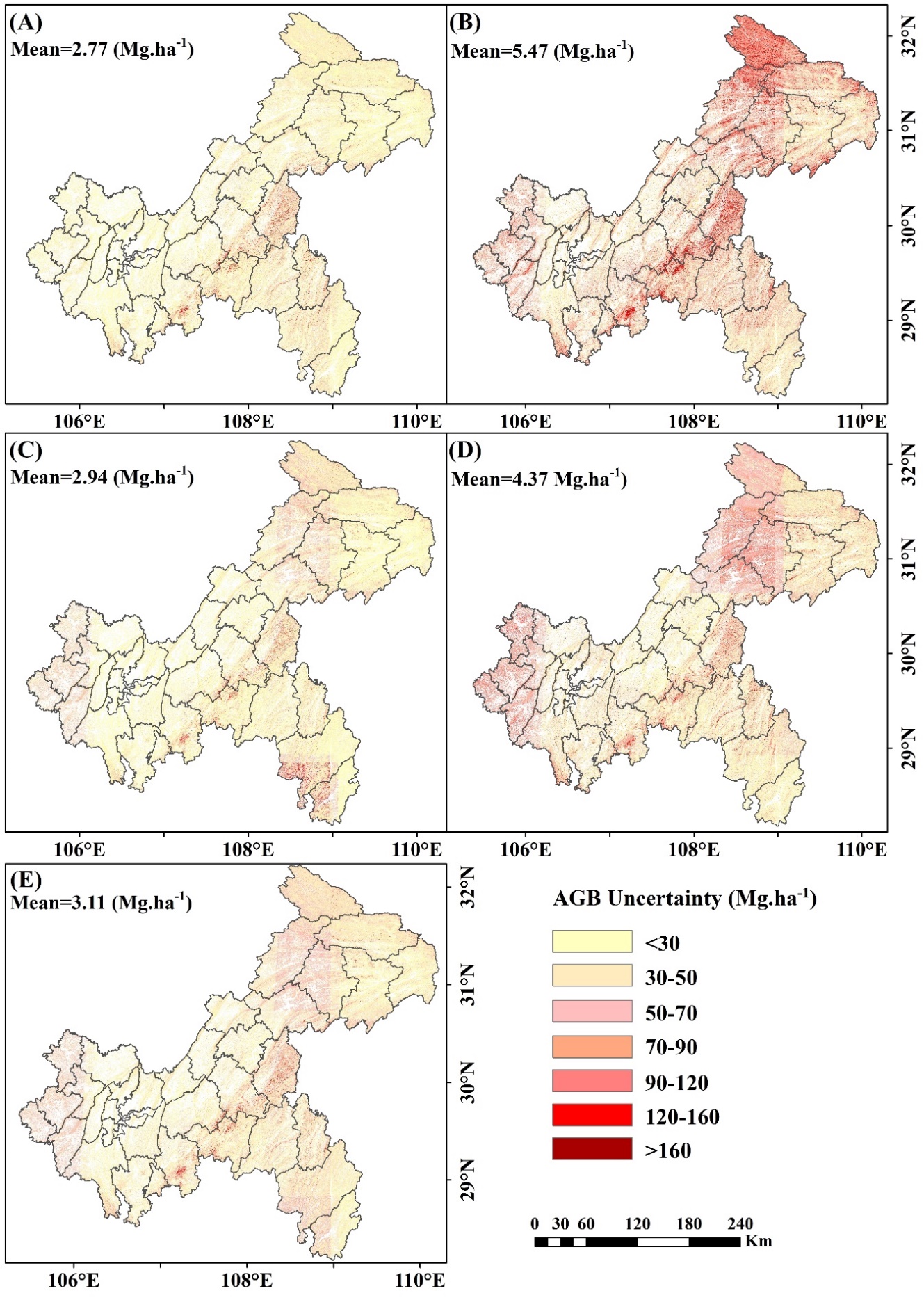


**Supplementary Figure S2.** Spatial distribution of uncertainty of AGB excluding under-threshold trees. (A) RF; (B) XgBoost; (C) CatBoost; (D) SVM; and (E) Stacked ensemble model.

## Supplementary Tables

**Supplementary Table S1**. Parameters of “Other” Groups for Plot AGB estimation.

| **Groups** | **Tree species** |
| --- | --- |
| Other pines | *Pinus thunbergii, Pinus deodara* |
| Other hardwood broadleaves | *Cinnamomum japonicum Sieb., Celtis sinensis Pers., Castanea mollissima, Zelkova serrata, Ligustrum lucidum, Liriodendron chinense, Bischofia polycarpa, Albizia julibrissin Durazz, Toona sinensis, Toona ciliata Roem., Bischofia javanica, Amygdalus mira, Ginkgo biloba, Cinnamomum wilsonii Gamble, Fraxinus chinensis Roxb, Pistacia chinensis Bunge, Swida wilsoniana, Ilex chinensis, Sophora xanthantha, Corylus heterophylla, Rhododendron simsii Planch, Pyracantha fortuneana* |
| Other softwood broadleaves | *Litsea cubeba, Lagerstroemia indica, Symplocaceae Symplocos, Erythrina variegata, Acacia mearnsii De Wilde, Platycarya strobilacea, Camptotheca acuminata Decne, Koelreuteria paniculata Laxm., Cerasus serrulata, Alnus cremastogyne, Acer mono Maxim, Ficus microcarpa, Ficus virens Aiton var. Sublanceolata, Citrus reticulata Blanco, Pyrus spp, Prunus salicina, Malus pumila Mill., Armeniaca vulgaris Lam., Ziziphus jujuba Mill., Crataegus pinnatifida Bge., Diospyros kaki, Litchi chinensis Sonn., Dimocarpus longan Lour., Cerasus pseudocerasus, Myricaceae Myrica, Citurs sinensis L.Osbeck, Malus spectabilis, Eriobotrya japonica, Camellia oleifera Abel., Olea europaea L., Camellia sinensis, Illicium verum Hook. f., Cinnamomum cassia Presl, Osmanthus fragrans, Lindera glauca, Eucommia ulmoides Oliver, Magnolia officinalis Rehd. et Wils., Clausena lansium, Toxicodendron vernicifluum, Vernicia fordii, Sapium sebiferum, Morus alba, Halimodendron Fisch., Syzygium aromaticum, Viburnum dilatatum, Amorpha fruticosa, Lonicera japonica, Cotoneaster, Berberidaceae Berberis, Eurya japonica Thunb., Sophora davidii, Elaeagnus pungens, Cotinus coggygria Scop., Coriaria nepalensis, Rosa multifolora Thunb, Aralia chinensis* |

**Supplementary Table S2**. Range of grid search parameter.

| **Model** | **Parameters** | **Range** |
| --- | --- | --- |
| RF | n_estimators | 100, 200, 300, 400, 500 |
|  | max_depth | 3, 6, 9, 12 |
| XgBoost | nrounds | 100, 200, 300,400,500,600 |
|  | max_depth | 3, 6, 9 |
|  | eta | 0.01, 0.1, 0.3 |
| CatBoost | iterations | 100, 200, 300,400,500,600 |
|  | max_depth | 3, 6, 9 |
|  | learning_rate | 0.01, 0.1, 0.3 |
| SVM | Cost | 0.1, 1, 10, 100 |
|  | sigma | 0.001,0.01, 0.1, 1 |

**Supplementary Table S3**. The results of CV validation and EV validation of the meta-model.

| **Stacked ensemble model** | **CV** | | | **EV** | | |
| --- | --- | --- | --- | --- | --- | --- |
|  | **R^2^** | **MAE**  **(**Mg·ha^-1^**)** | **RMSE**  **(**Mg·ha^-1^**)** | **R^2^** | **MAE**  **(**Mg·ha^-1^**)** | **RMSE**  **(**Mg·ha^-1^**)** |
| LM | 0.66 | 19.39 | 25.06 | 0.68 | 19.12 | 25.41 |
| KNN | 0.77 | 15.85 | 21.15 | 0.65 | 19.41 | 26.35 |
| RR | 0.65 | 19.61 | 25.58 | 0.68 | 18.87 | 25.45 |
| Entropy weight | 0.65 | 19.81 | 26.09 | 0.67 | 18.86 | 25.85 |

**Supplementary Table S4**. Comparison of model performance.

| **Model** | **Performance** | | |
| --- | --- | --- | --- |
|  | **R2** | **MAE**  **(**Mg·ha^-1^**)** | **RMSE**  **(**Mg·ha^-1^**)** |
| ANN | 0.53 | 23.35 | 31.13 |
| LM | 0.56 | 21.83 | 29.40 |
| RR | 0.55 | 22.13 | 29.23 |
